# Hormonal metabolic burden in diabetic retinopathy: the diabetic retina as an endocrine-responsive target organ

**DOI:** 10.3389/fendo.2026.1899237

**Published:** 2026-08-31

**Authors:** Jing Chen, Ling Zhang

**Affiliations:** Department of Ophthalmology, People’s Hospital of Leshan, Leshan, Sichuan, China

**Keywords:** diabetic macular edema, diabetic retinopathy, endocrine-responsive retina, GH/IGF-1 axis, hormonal metabolic burden, HPA axis, sex steroids, thyroid hormone

## Abstract

Diabetic retinopathy (DR) is usually interpreted as a complication of chronic hyperglycemia and vascular injury. This model remains fundamental, but it does not fully explain early, rapid, or treatment-resistant disease in some patients. Puberty, pregnancy, menopause, obstructive sleep apnea, thyroid dysfunction, chronic stress, and rapid improvement in glycemic control are clinical settings in which retinal disease may change despite standard risk assessment. We propose the Hormonal Metabolic Burden (HMB) hypothesis, which views the diabetic retina as an endocrine-responsive neural and vascular tissue. In this model, growth hormone/insulin-like growth factor-1 (GH/IGF-1), hypothalamic–pituitary–adrenal (HPA)–glucocorticoid signaling and related glucocorticoid receptor/mineralocorticoid receptor (GR/MR) balance, thyroid hormone, and sex steroid signaling act on retinal neurons, Müller glia, endothelial cells, pericytes, and retinal pigment epithelial cells through receptor- and enzyme-mediated pathways. These axes may converge with hyperglycemia on oxidative stress, nuclear factor kappa B (NF-κB)-mediated inflammation, vascular endothelial growth factor (VEGF) signaling, blood-retinal barrier (BRB) dysfunction, neurodegeneration, and metabolic memory. HMB is proposed as an additional research layer rather than a replacement for established DR risk assessment, and it should not be used to justify broad hormone testing or off-label endocrine treatment. This hypothesis generates testable predictions: multi-axis endocrine profiling should improve risk prediction beyond standard variables; prespecified endocrine-retinal profiles characterized by greater magnitude, persistence, or co-occurrence of selected axis-specific disturbances should be associated with distinct optical coherence tomography (OCT) or optical coherence tomography angiography (OCTA) trajectories; persistent or suboptimally responsive diabetic macular edema (DME) despite an adequate course of anti-VEGF therapy should be enriched for selected endocrine signals; and later-stage biomarker-selected studies should test whether matched endocrine correction has greater retinal effects than unselected intervention. It is a research-oriented model for selected phenotypes, including rapid nonproliferative diabetic retinopathy, persistent or suboptimally responsive DME, pubertal proliferative disease, postmenopausal acceleration, thyroid-linked disease, and diabetic retinopathy with sleep apnea. If validated, this framework may support more focused endocrine-retinal research and improve the design of precision prevention studies in selected DR phenotypes.

## 1 Introduction

Diabetic retinopathy (DR) remains one of the most common and serious microvascular complications of diabetes. Global estimates suggest that more than 100 million people have DR, and this burden is expected to increase further by 2045 (1). DR is clinically important because it affects working-age adults, increases healthcare costs, and often requires repeated specialist visits. It is also a visible marker of systemic vascular injury. Current treatments, including anti-vascular endothelial growth factor (VEGF)-targeted therapy, laser photocoagulation, vitrectomy, and corticosteroid implants, have improved the management of diabetic macular edema (DME) and proliferative DR. Modern imaging and screening programs have also improved early detection. However, these approaches mainly treat leakage, neovascularization, and structural complications after retinal injury has already developed. They do not fully explain why some patients develop early, aggressive, or treatment-resistant disease (2–6). The standard pathogenic model of DR remains centered on chronic hyperglycemia. This model is essential and supported by landmark clinical trials showing that intensive glycemic and blood pressure control reduces the risk of DR onset and progression (7, 8). However, a glucose-centered model does not explain all clinical patterns. In clinical practice, some patients progress despite apparently acceptable glycemic control, whereas others show rapid retinal changes during puberty, pregnancy, menopause, major weight loss, sleep apnea, chronic stress, thyroid disease, or rapid improvement in glycemic control. Early worsening after rapid glycemic improvement is a clear example. A systematic review showed that early worsening can occur after substantial glycated hemoglobin (HbA1c) reduction, particularly in patients with long-standing poor control and more severe baseline retinopathy (9). These observations do not weaken the role of glucose. Instead, they suggest that the diabetic retina responds to a broader metabolic and endocrine environment.This article proposes the Hormonal Metabolic Burden (HMB) hypothesis. HMB refers to a multidimensional, axis-specific, time-dependent, and phenotype-dependent endocrine-retinal burden profile generated by several hormonal axes. Its biological meaning depends on the magnitude, duration, timing, co-occurrence, dominant axis, retinal stage, and metabolic context of endocrine disturbances, rather than on the number of abnormal hormone values. The main axes considered in this framework are growth hormone/insulin-like growth factor-1 (GH/IGF-1), hypothalamic–pituitary–adrenal (HPA)–glucocorticoid signaling and the related glucocorticoid receptor/mineralocorticoid receptor (GR/MR) balance, thyroid hormone signaling, and sex steroid signaling. The central idea is that several mild or subclinical hormonal disturbances may have limited effects in isolation but may act together to lower retinal reserve and amplify diabetic injury. These endocrine signals may converge with hyperglycemia on shared retinal pathways, including oxidative stress, inflammation, VEGF signaling, blood-retinal barrier dysfunction, neurodegeneration, and metabolic memory. This is a Hypothesis and Theory article, not a clinical guideline. We do not propose broad hormone testing or off-label endocrine treatment for all patients with diabetes. Instead, we propose HMB as a research-oriented framework for understanding residual retinal risk and treatment heterogeneity in selected phenotypes, such as rapid DR despite reasonable systemic control, persistent or suboptimally responsive DME despite an adequate course of anti-VEGF therapy, pubertal or young-onset proliferative DR, postmenopausal acceleration, thyroid-linked disease, and DR with untreated sleep apnea. The clinical rationale for this framework is practical. Retina specialists often encounter patients whose disease course appears disproportionate to their standard risk profile. These atypical or disproportionate phenotypes provide the clinical entry point for testing the HMB framework.

The conceptual novelty of HMB does not lie in proposing that any single endocrine pathway independently causes DR, because each of the candidate axes has previously been investigated in isolation. Rather, HMB reframes these signals as a multidimensional, retina-centered susceptibility profile whose biological meaning depends on the magnitude, duration, timing, and co-occurrence of endocrine disturbances, as well as on retinal stage, cellular compartment, and the surrounding metabolic environment. Accordingly, “burden” does not refer simply to the number of hormone values outside their reference ranges or to an unvalidated additive score. The framework differs from a catalogue of known endocrine modifiers in four respects. First, it treats the diabetic retina as an endocrine-responsive target organ with local receptor-, enzyme-, and cell-specific signaling. Second, it proposes that modest disturbances across axes may interact additively, synergistically, hierarchically, or sequentially to lower retinal reserve. Third, it adopts a phenotype-first approach, in which an atypical retinal course defines the endocrine-retinal research question rather than prompting indiscriminate hormonal testing. Fourth, it is explicitly falsifiable: HMB should provide predictive or mechanistic information beyond established DR risk factors, and the hypothesis would be weakened if multi-axis profiling failed to improve longitudinal prediction, identify reproducible retinal phenotypes, or demonstrate context-dependent interactions. HMB is therefore intended as a complementary mechanistic layer within established hyperglycemic, inflammatory, neurovascular, and metabolic-memory models of DR, rather than as a replacement for these models or as a validated clinical score. Several established models of DR remain essential for understanding retinal injury, including hyperglycemia-mediated microvascular damage, neurovascular unit dysfunction, oxidative stress and metabolic memory, and clinical multifactorial risk prediction. The HMB framework is not intended to compete with these models. Instead, it adds a phenotype-first endocrine-retinal layer that asks whether multi-axis endocrine context can explain residual risk, treatment heterogeneity, and pathway-specific retinal vulnerability beyond standard variables. The conceptual relationship between HMB and established DR models is summarized in **Table 1**.

**Table 1:** Comparison of established diabetic retinopathy models with the Hormonal Metabolic Burden framework.

Framework	Primary explanatory focus	Main unit of analysis	Temporal or burden dimension	Interaction structure	What HMB adds
Classical hyperglycemia–microvascular model	Glucose-mediated vascular injury	Retinal microvasculature	Chronic exposure	Mostly downstream pathway convergence	Endocrine modulation of susceptibility under similar glycemic exposure
Neurovascular unit/inflammation model	Coupled neural, glial and vascular dysfunction	Retinal neurovascular unit	Early and progressive injury	Cellular cross-talk	Systemic and local endocrine signals acting on different neurovascular compartments
Oxidative stress/metabolic memory model	Persistent molecular injury after metabolic exposure	Mitochondrial and epigenetic pathways	Strong temporal memory	Pathway amplification	Candidate hormonal contribution to priming, amplification and persistence
Clinical multifactorial risk models	Prediction using HbA1c, BP, diabetes duration, kidney disease, and lipid variables	Patient-level clinical risk	Longitudinal risk	Usually additive statistical predictors	Axis-specific and interaction-based explanation of residual risk
HMB framework	Endocrine-retinal susceptibility and treatment heterogeneity	Phenotype-matched endocrine-retinal profile	Magnitude, duration, timing, co-occurrence, retinal stage, and memory	Additive, synergistic, hierarchical, or sequential; to be tested	A multi-axis, phenotype-first, target-organ framework with falsifiable predictions

BP, blood pressure; DR, diabetic retinopathy; HbA1c, glycated hemoglobin; HMB, Hormonal Metabolic Burden. These models are complementary rather than mutually exclusive. HMB is proposed as an additional endocrine-retinal mechanistic layer for explaining residual risk and treatment heterogeneity, rather than as a replacement for established DR models or as a validated clinical score.

In operational terms, the central HMB hypothesis is that, in selected diabetic retinal phenotypes, the magnitude, timing, co-occurrence, and interaction of GH/IGF-1 signaling, HPA–glucocorticoid signaling and related GR/MR balance, thyroid hormone signaling, and sex steroid signaling modify retinal reserve, disease trajectory, and treatment heterogeneity beyond conventional DR risk factors. HMB should therefore be positioned as a staged research framework rather than as a stage-specific clinical tool. Its immediate application is phenotype-guided research cohort enrichment and longitudinal testing of incremental predictive value, not routine clinical screening, diagnosis, hormone-panel testing, routine ocular treatment selection, or endocrine intervention. Early or rapidly progressive DR phenotypes are used to study residual risk and future precision-prevention hypotheses, whereas persistent or suboptimally responsive DME phenotypes are used to study treatment-response heterogeneity. Prevention-oriented or biomarker-selected interventional studies should be regarded as later-stage possibilities that require prospective replication, mechanistic validation, retinal endpoint evidence, and systemic safety assessment.

## 2 The diabetic retina as an endocrine-responsive target organ

The retina is not only a vascular tissue. It is a highly metabolic neural tissue with strict barrier control and close communication among neurons, glia, vessels, and the retinal pigment epithelium (RPE). The retinal neurovascular unit includes endothelial cells, pericytes, Müller glia, astrocytes, microglia, photoreceptors, bipolar cells, ganglion cells, and RPE. These cells share the same diabetic environment, but they do not respond to systemic signals in the same way. Each compartment may interpret hormonal and metabolic signals through its own receptors, enzymes, and stress-response pathways. This cellular diversity provides the biological basis for viewing the diabetic retina as an endocrine-responsive target organ.

Several endocrine-related systems are present in retinal tissue. Retinal insulin signaling has been described in neurons, vascular cells, and RPE. Reviews of retinal insulin resistance and local retinal insulin biology suggest that insulin signaling is not only a systemic background variable but may directly influence retinal metabolism, barrier function, and neuronal survival (10, 11). This is important because impaired insulin signaling can also modify IGF-1 signaling, inflammatory responses, and endothelial resilience.

Thyroid hormone signaling is another relevant pathway. Thyroid hormone receptors support retinal development and adult photoreceptor function, while local conversion of thyroxine (T4) to triiodothyronine (T3) through deiodinase activity allows the retina to regulate active thyroid hormone exposure at the tissue level (12). In diabetes, hyperglycemia and inflammation may disturb this local control and reduce retinal metabolic reserve.

Corticosteroid signaling also has direct retinal relevance. GR is active in retinal glia, especially Müller cells. A 2024 experimental study identified GR as a key regulator of the Müller cell response to diabetic conditions. Loss of GR signaling was associated with gliosis, whereas restoration of GR activity improved retinal function in experimental models (13).

This finding helps explain an important clinical distinction. Intravitreal corticosteroids can reduce DME by suppressing local inflammation and stabilizing the blood-retinal barrier, but chronic systemic cortisol stress may produce different effects depending on dose, duration, receptor balance, circadian rhythm, and local retinal state. MR signaling may further contribute to retinal inflammation, vascular dysfunction, and edema-related phenotypes.

**Figure 1:**
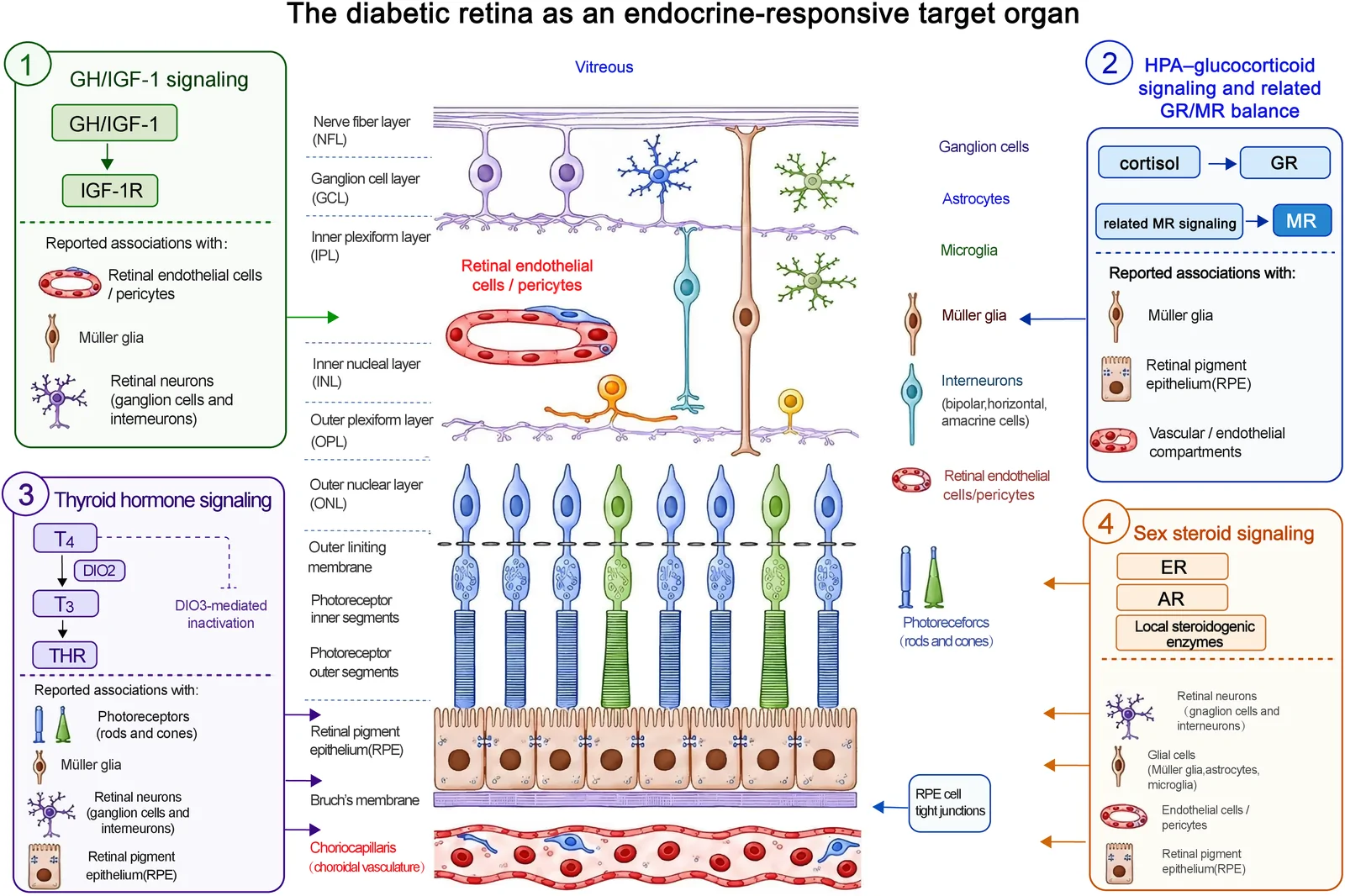
The diabetic retina as an endocrine-responsive target organ. The diabetic retina contains multiple endocrine-responsive compartments within the retinal neurovascular unit. GH/IGF-1 signaling may act through IGF-1R in retinal endothelial cells and pericytes, Müller glia, and retinal neurons. HPA–glucocorticoid signaling may act through cortisol–GR pathways, whereas related retinal MR signaling may act through MR in Müller glia, the retinal pigment epithelium, and vascular/endothelial compartments. Thyroid hormone signaling may involve thyroid hormone receptors, DIO2-mediated local conversion of T4 to T3, and DIO3-mediated thyroid hormone inactivation in photoreceptors, Müller glia, retinal neurons, and the retinal pigment epithelium. Sex steroid signaling may involve estrogen receptors, androgen receptors, and local steroidogenic enzymes in retinal neuronal, glial, endothelial, and retinal pigment epithelial compartments. Collectively, these receptor and enzyme systems provide a biological basis for endocrine modulation of oxidative stress, inflammation, VEGF signaling, blood–retinal barrier integrity, and neurodegeneration in diabetic retinopathy, although these downstream processes are not depicted in the figure. Arrows indicate reported hormone–receptor associations, enzyme-mediated conversions, or receptor-, enzyme-, and cell-compartment associations within the retinal neurovascular unit; they do not depict hypothesized cross-axis HMB interactions. AR, androgen receptor; DIO2, type 2 iodothyronine deiodinase; DIO3, type 3 iodothyronine deiodinase; ER, estrogen receptor; GCL, ganglion cell layer; GH, growth hormone; GR, glucocorticoid receptor; HMB, Hormonal Metabolic Burden; HPA, hypothalamic–pituitary–adrenal; IGF-1, insulin-like growth factor 1; IGF-1R, insulin-like growth factor 1 receptor; INL, inner nuclear layer; IPL, inner plexiform layer; MR, mineralocorticoid receptor; NFL, nerve fiber layer; ONL, outer nuclear layer; OPL, outer plexiform layer; RPE, retinal pigment epithelium; T3, triiodothyronine; T4, thyroxine; THR, thyroid hormone receptor; VEGF, vascular endothelial growth factor.

Sex steroid signaling provides another layer of endocrine responsiveness. Estrogen receptors, androgen-related pathways, and local steroidogenic enzymes have been reported in the retina and RPE (14, 15). Experimental and translational models suggest that sex steroid signaling may affect oxidative stress, glial activation, endothelial barrier function, and neuronal survival. A human inner blood-retinal barrier-on-chip model can reproduce early DR-like changes, and estradiol has been shown to affect Müller glia and endothelial cell responses under hyperglycemic and advanced glycation end-product conditions (16, 17). These findings support the idea that sex steroid status may modify diabetic retinal vulnerability, although it should not be interpreted as evidence for broad hormone therapy. These findings provide the rationale for **Figure 1**.

Taken together, these receptor and enzyme systems support the concept that the diabetic retina can respond directly to systemic and local endocrine signals (**Figure 1**). This does not mean that every hormonal abnormality causes DR. Rather, endocrine signals may change the threshold at which diabetic retinal injury begins, progresses, or becomes resistant to standard treatment. Three retinal features are especially relevant. First, the retina has high energy demand. Photoreceptors, ganglion cells, and Müller glia require continuous metabolic support, and even a modest decline in mitochondrial reserve may increase susceptibility to oxidative injury. Second, the retina depends on barrier integrity. Hormonal signals that affect endothelial junctions, RPE transport, Müller cell water handling, or pericyte stability may influence edema and neurovascular stress. Third, the retina is compartmentalized. Serum hormone levels may not fully reflect retinal tissue exposure because thyroid hormone can be locally converted, corticosteroids can act through both GR and MR, IGF-1 signaling can be redirected by insulin resistance, and sex steroids may act locally in retinal and RPE compartments. This endocrine-responsive structure is the cellular foundation of the HMB hypothesis. HMB should therefore not rely only on blood-based endocrine markers. It should be tested by combining systemic biomarkers, retinal imaging, and experimental models. The most convincing evidence will come when endocrine profiles, optical coherence tomography (OCT) or optical coherence tomography angiography (OCTA) phenotypes, and retinal model systems point to the same axis-specific pattern of injury.

## 3 Core endocrine axes selected for the initial HMB framework

The initial HMB framework focuses on four core endocrine axes: GH/IGF-1 signaling, HPA–glucocorticoid signaling and the related GR/MR balance, thyroid hormone signaling, and sex steroid signaling. These axes were selected not because they are the only endocrine systems relevant to DR, but because they meet several criteria for an initial, testable endocrine-retinal framework. First, each represents a relatively organized endocrine axis or receptor–enzyme signaling system rather than a single isolated circulating marker. Second, each has a plausible basis for direct retinal responsiveness through receptors, local hormone metabolism, or cell-specific signaling within the neurovascular unit. Third, each corresponds to recognizable clinical endocrine states or transition periods, such as puberty, pregnancy, menopause, thyroid dysfunction, chronic stress, sleep disruption, or altered corticosteroid receptor balance. Fourth, each has both clinical and mechanistic evidence that can be provisionally matched to retinal phenotypes, including proliferative activity, recurrent or persistent edema, early neurodegeneration, vascular leakage, or disproportionate OCT/OCTA change. Fifth, each can be measured or contextualized longitudinally in clinical research cohorts, allowing axis-specific hypotheses to be tested rather than relying on a nonspecific endocrine risk label.

Other endocrine-related systems may also influence diabetic retinal disease. Adipokines such as leptin and adiponectin, incretin biology including glucagon-like peptide-1 (GLP-1) signaling, vitamin D–parathyroid hormone (PTH)–calcium/phosphate metabolism, fibroblast growth factor 21 (FGF21) and related liver–adipose–retina metabolic signals, and other emerging endocrine mediators may all be relevant to DR biology. These systems are not excluded because they are biologically unimportant. Rather, they are placed outside the initial core HMB framework because their current evidence differs in mechanistic maturity, clinical interpretability, treatment-related confounding, or suitability for axis-specific longitudinal profiling. They should be considered candidate extension modules for future versions of HMB once their retinal phenotypes, measurement strategy, and incremental value beyond standard DR variables are more clearly defined. This boundary is important because HMB is intended to be a disciplined, phenotype-matched research framework rather than an open-ended list of all possible endocrine or metabolic biomarkers.

**Figure 2:**
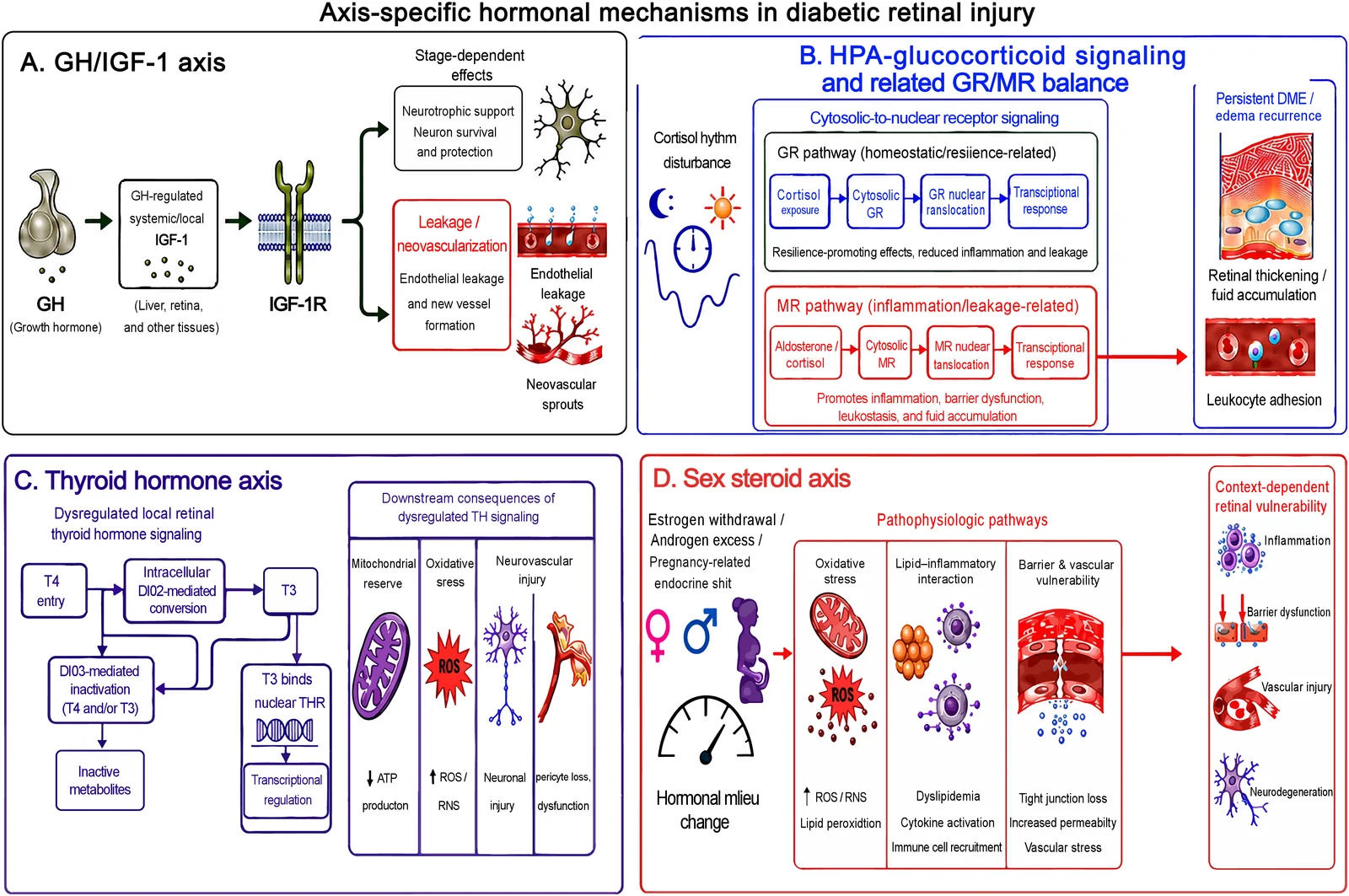
Axis-specific hormonal mechanisms in diabetic retinal injury. The four endocrine axes included in the HMB framework may affect the diabetic retina through partly distinct but overlapping mechanisms. **(A)** The GH/IGF-1 axis may act through IGF-1R signaling and downstream PI3K/Akt, HIF-1α, NF-κB, AP-1, and VEGF pathways, with stage-dependent neurotrophic or angiogenic effects. **(B)** The HPA–glucocorticoid signaling module and related GR/MR balance may involve cortisol rhythm disturbance, protective glucocorticoid receptor signaling in Müller glia, and mineralocorticoid receptor-driven inflammation, leakage, leukostasis, and barrier dysfunction. MR signaling is shown as a related retinal effector pathway rather than as a direct HPA output. **(C)** The thyroid hormone axis may act through local T4-to-T3 conversion, DIO2 activity, thyroid hormone receptor signaling, mitochondrial reserve, oxidative stress, neurodegeneration, and pericyte vulnerability. **(D)** The sex steroid axis may involve estrogen withdrawal, androgen excess, pregnancy-related endocrine shifts, oxidative stress, lipid-inflammatory interactions, and barrier and vascular vulnerability. These pathways may converge on retinal inflammation, blood-retinal barrier dysfunction, vascular injury, neurodegeneration, and treatment heterogeneity in diabetic retinopathy. Solid arrows indicate evidence-informed or mechanistically plausible axis-to-pathway links. Downstream retinal phenotypes are proposed context-dependent implications that require validation in endocrine-retinal cohorts and experimental models. Akt, protein kinase B; AP-1, activator protein-1; AR, androgen receptor; ATP, adenosine triphosphate; BRB, blood-retinal barrier; DIO2, type 2 deiodinase; DIO3, type 3 deiodinase; DME, diabetic macular edema; ER, estrogen receptor; GH, growth hormone; GR, glucocorticoid receptor; HIF-1α, hypoxia-inducible factor-1 alpha; HMB, Hormonal Metabolic Burden; HPA, hypothalamic–pituitary–adrenal; IGF-1, insulin-like growth factor-1; IGF-1R, insulin-like growth factor-1 receptor; MR, mineralocorticoid receptor; NF-κB, nuclear factor kappa B; PI3K, phosphoinositide 3-kinase; RNS, reactive nitrogen species; ROS, reactive oxygen species; T3, triiodothyronine; T4, thyroxine; THR, thyroid hormone receptor; VEGF, vascular endothelial growth factor.

Within this structure, conventional DR risk factors remain the foundational layer, including HbA1c and its trajectory, blood pressure, diabetes duration, kidney disease, lipid status, and baseline retinopathy severity. The four selected endocrine axes form the initial HMB core. Clinical and physiological states such as puberty, pregnancy, menopause, obesity, obstructive sleep apnea, medication changes, chronic stress, and rapid glycemic improvement are best viewed as contextual gates that may determine which axis becomes biologically dominant in a given patient, rather than as separate HMB axes by themselves. The four core axes may affect the diabetic retina through partly distinct but overlapping molecular pathways (**Figure 2**).

### 3.1 GH/IGF-1 axis: stage-dependent angiogenic and neurotrophic signaling

#### 3.1.1 Clinical observation

The GH/IGF-1 axis has one of the longest histories in diabetic retinopathy research. In 1953, Poulsen reported regression of severe diabetic retinopathy after pituitary infarction (18). Later observations linked hypopituitarism or growth hormone deficiency with low rates of retinopathy despite diabetes (19). Surgical pituitary ablation was also used historically in severe proliferative diabetic retinopathy, and the ocular response was related to the completeness of pituitary ablation (20, 21). These studies are not a basis for modern treatment, but they remain important because they show that a systemic endocrine signal can influence retinal disease activity. Puberty provides another clinical example. Diabetic retinopathy may accelerate in adolescents with type 1 diabetes during a period of increased GH pulsatility and changing IGF-1 biology, and this pattern is not fully explained by HbA1c alone (22). Acromegaly gives a further example, because excess GH and IGF-1 may resemble or aggravate diabetic retinal injury, especially when glucose intolerance or overt diabetes is present (23).

#### 3.1.2 Molecular and cellular basis

IGF-1 can act through IGF-1 receptor signaling in retinal endothelial cells, glia, and neurons. In the diabetic retina, its effect is likely to be stage-dependent. In an early or low-stress state, insulin/IGF-1 signaling may support neuronal survival, protein kinase B (Akt)-mediated trophic signaling, and retinal metabolic resilience. In a hypoxic, inflamed, or ischemic retina, the same axis may contribute to vascular leakage, neovascularization, and fibrovascular activity. Mechanistically, insulin-like growth factor-1 receptor (IGF-1R) signaling may converge on phosphoinositide 3-kinase (PI3K)/Akt, hypoxia-inducible factor-1 alpha (HIF-1α), nuclear factor kappa B (NF-κB), activator protein-1 (AP-1), and VEGF-related pathways. Experimental data show that increased ocular IGF-1 can produce diabetes-like retinal vascular changes, including vascular leakage and capillary pathology (24, 25). Hyperglycemia may also alter downstream signaling. When insulin receptor substrate signaling is impaired, IGF-1R activation may be redirected toward pathways that favor endothelial dysfunction and retinal vascular injury (26).

#### 3.1.3 Research implication for HMB

For HMB, the GH/IGF-1 axis should be treated as a conditional signal rather than a single linear risk factor. Its retinal effect may depend on age, diabetes type, pubertal status, insulin resistance, ischemia, inflammation, and baseline retinal stage. This may explain why older somatostatin analog studies were difficult to interpret. Somatostatin analogs suppress GH/IGF-1 and may also act on retinal somatostatin receptors. Earlier studies suggested benefit in selected DR populations, and later safety analyses did not identify major unexpected toxicity, but these studies predated modern DME management and did not select patients by IGF-1 burden or OCT phenotype (27–29). More recent work on lanreotide in persistent DME reported clinical and mechanistic signals, including effects on VEGF and oxidative stress-related apoptosis in RPE cells (30). These findings support a more focused research question: does GH/IGF-1 modulation benefit patients whose retinal phenotype suggests active IGF-1 involvement? Future studies should consider pubertal proliferative DR, acromegaly-associated retinal disease, high age-adjusted IGF-1, altered IGF bioactivity, and persistent DME with growth-factor-related features as enrichment phenotypes.

### 3.2 HPA–glucocorticoid signaling and related GR/MR balance: cortisol-related retinal stress

#### 3.2.1 Clinical observation

HPA–glucocorticoid signaling is often altered in type 2 diabetes, obesity, sleep disruption, depression, chronic stress, and obstructive sleep apnea. Patients may show increased cortisol burden, a flatter diurnal rhythm, or impaired suppression. In the HMB framework, this axis is discussed together with retinal GR/MR balance because corticosteroid receptor signaling, rather than circulating cortisol alone, may be the biologically relevant retinal effector. Systemically, cortisol can worsen insulin resistance, and insulin resistance may further activate stress-related pathways (31, 32). This creates a systemic loop that may reach the retina. The clinical interpretation of corticosteroid biology in DR must be careful. Intravitreal corticosteroids reduce DME in many patients by suppressing inflammation, reducing leakage, and stabilizing the blood-retinal barrier. This local therapeutic effect does not mean that chronic systemic cortisol stress is protective. In the retina, the effect of corticosteroid signaling likely depends on receptor balance, tissue compartment, exposure duration, circadian rhythm, and diabetic retinal state.

#### 3.2.2 Molecular and cellular basis

GR and MR signaling appear to have different retinal implications. GR signaling may be protective in specific retinal cells, especially Müller glia. Müller cells maintain potassium homeostasis, glutamate handling, water transport, barrier support, and metabolic support for neurons. They also become reactive in diabetes. A 2024 experimental study identified GR as a key regulator of the Müller cell response to diabetic conditions, and loss of GR signaling was associated with gliosis and retinal dysfunction (13). Other work links glucocorticoid-induced TSC22 domain family member 3 (TSC22D3) to HIF-1α and galectin-1 pathways in the diabetic retina (33). These findings suggest that local GR activity may help restrain harmful glial programs. MR signaling is included here as a related retinal corticosteroid effector module, not to imply that aldosterone is primarily regulated by the HPA axis. MR is present in retinal tissues, and cortisol- or aldosterone-related MR activation may promote inflammation, vascular dysfunction, and edema-related phenotypes. Reviews and experimental studies link MR biology to retinal inflammation, leakage, and vascular injury (34–37). A 2024 transcriptomic study showed that aldosterone exposure or MR overexpression produced a low-grade inflammatory retinal program distinct from GR signaling. In rodent models of diabetic and neovascular retinopathy, finerenone reduced vascular injury and increased regulatory T-cell-related signals (38).

#### 3.2.3 Research implication for HMB

HPA–glucocorticoid signaling and related GR/MR balance contribute to HMB through at least three mechanisms: systemic insulin resistance, reduced Müller cell resilience through impaired GR signaling, and MR-driven inflammation or leakage. Sleep apnea provides an important clinical setting in which these mechanisms may be tested. Obstructive sleep apnea is common in diabetes and can cause intermittent hypoxia, sympathetic activation, sleep fragmentation, and cortisol rhythm disturbance. A large real-world study found that sleep apnea was associated with worse DR progression and more retinal intervention (39). A randomized clinical trial showed that continuous positive airway pressure (CPAP) reduced retinal lesion progression in patients with sleep apnea and nonproliferative DR (40). A Danish registry study did not show the same direction for vision-threatening DR, but it had limitations, including the lack of detailed glycemic and blood pressure data (41). A 2025 genetic study of sleep traits and DR also supports further evaluation of sleep physiology as a possible modifying pathway (42). For HMB research, a single morning cortisol level is unlikely to be enough. Better designs should capture sleep apnea status, oxygen desaturation, CPAP adherence, cortisol rhythm, medication exposure, kidney disease, blood pressure, potassium, and MR antagonist exposure. The most relevant retinal phenotypes may include recurrent or persistent DME, choroidal vascular change, glial activation, and rapid progression that is not explained by HbA1c alone.

### 3.3 Thyroid hormone axis: local T3 bioavailability and retinal metabolic reserve

#### 3.3.1 Clinical observation

Thyroid hormone signaling is essential for retinal development and adult retinal function. In clinical studies, thyroid status has been linked to DR, although the evidence is not uniform. A 2020 study in patients with type 2 diabetes and normal thyroid function found an association between normal-range thyroid hormones and DR (43). A 2023 two-sample Mendelian randomization study examined thyroid traits and diabetic microvascular complications and found stronger evidence for diabetic kidney disease than for DR, which is an important caution (44). More recent studies have strengthened the signal for free triiodothyronine (FT3). A 2025 longitudinal cohort and Mendelian randomization study reported that higher free T3 was associated with a lower risk of DR onset or progression (45). A 2026 study in euthyroid patients with type 2 diabetes found that DR was associated with lower FT3, lower resting metabolic rate, and higher systemic immune-inflammation index (46). These findings do not prove that T3 therapy prevents DR. They suggest that low-normal FT3 may identify a low-reserve metabolic and inflammatory phenotype.

#### 3.3.2 Molecular and cellular basis

The thyroid axis is important because serum thyroid tests may not fully reflect retinal thyroid hormone exposure. In the mature retina, local T3 bioavailability may depend on thyroid hormone transport, thyroid hormone receptors, deiodinase activity, and mitochondrial response. Local T4-to-T3 conversion allows retinal tissue to regulate active thyroid hormone exposure at the tissue level (12). In diabetes, hyperglycemia, inflammation, kidney disease, and systemic metabolic stress may disturb this local control. Low T3 activity may reduce mitochondrial reserve and make photoreceptors, Müller glia, neurons, and pericytes less able to tolerate hyperglycemic stress. In contrast, overt hyperthyroidism may increase oxidative stress, hemodynamic stress, and VEGF-related signaling. Therefore, both insufficient and excessive thyroid hormone activity may be harmful in different contexts.

#### 3.3.3 Research implication for HMB

For HMB, thyroid biology should be treated as a retinal resilience axis rather than a simple hypothyroidism-versus-euthyroidism category. A patient with thyroid-stimulating hormone (TSH) and free thyroxine (FT4) within the reference range may still have low-normal FT3, reduced metabolic reserve, or increased inflammatory burden. Future studies should separate overt thyroid disease, subclinical hypothyroidism, and low-normal FT3 phenotypes. They should also analyze thyroid markers both categorically and continuously. The most relevant retinal phenotypes may include early or rapid nonproliferative diabetic retinopathy (NPDR), inner retinal thinning, OCTA vessel density loss, and neurodegeneration that appears disproportionate to the standard risk profile. Interventional interpretation must remain cautious. Treating biochemical hypothyroidism is standard endocrine care, but treating low-normal FT3 only to protect the retina is not currently evidence-based. A future trial should begin with patients who already have a clinical endocrine indication or a clearly defined subclinical thyroid phenotype, and should include retinal endpoints, systemic safety, heart rhythm, bone risk, body weight, and symptoms.

### 3.4 Sex steroid axis: estrogenic support and context-dependent retinal risk

#### 3.4.1 Clinical observation

The retina is not sex-neutral. Sex steroid biology may help explain why some DR phenotypes change during puberty, pregnancy, menopause, polycystic ovary syndrome (PCOS), hypogonadism, or other states of altered gonadal hormone signaling. These settings should not be grouped under a single “female hormone” or “male hormone” explanation. Pregnancy may worsen DR despite high estrogen levels because it also involves major changes in blood volume, placental hormones, glycemia, blood pressure, and IGF-1-related biology. Puberty combines sex steroid changes with GH/IGF-1 activation and metabolic instability. Menopause reduces estrogenic support and is accompanied by changes in lipid biology, body composition, vascular tone, and inflammation. Polycystic ovary syndrome combines androgen excess, insulin resistance, obesity risk, and metabolic inflammation.

#### 3.4.2 Molecular and cellular basis

Estrogen receptors, androgen-related pathways, and local steroidogenic enzymes are present in ocular tissues, including the retina and RPE (14, 15). Experimental data suggest that estrogenic signaling can support vascular tone, reduce oxidative stress, and protect retinal neurons, glia, and barrier cells. A 2025 estradiol study in hyperglycemic Müller glia and endothelial systems showed lower reactive oxygen species and improved barrier resistance with estradiol exposure (17). These data support a protective biological signal, but they should not be overinterpreted as evidence for broad estrogen therapy in DR. Sex steroid effects are context-dependent and may be modified by age, diabetes type, lipids, insulin resistance, pregnancy status, menopausal status, and vascular risk.

#### 3.4.3 Research implication for HMB

For HMB, sex steroid status should be recorded as a biological context that modifies retinal vulnerability rather than as a direct treatment target. Recent clinical evidence supports this approach. A 2025 study in women with type 2 diabetes found that apolipoproteins and menopausal status interacted in relation to DR and proliferative DR risk (47). This does not prove that menopause causes DR, but it suggests that menopausal state can modify the relationship between systemic metabolic markers and retinal disease. Future studies should record menopausal status, age at menopause, hormone therapy exposure, estradiol context, sex hormone-binding globulin (SHBG), androgen markers, lipids, pregnancy status, PCOS status, and retinal imaging outcomes. Sex should not be treated only as a covariate. A 52-year-old postmenopausal woman, a 32-year-old woman with PCOS, a pregnant woman with type 1 diabetes, and a 70-year-old man with hypogonadism represent different endocrine states. In HMB research, these states should be pre-specified because they may interact with GH/IGF-1 signaling, HPA–glucocorticoid signaling and related GR/MR balance, thyroid hormone signaling, insulin resistance, and inflammatory pathways (**Table 2**).

**Table 2:** Evidence map for hormonal axes included in the HMB hypothesis.

Hormonal axis	Molecular evidence	Animal or model evidence	Human evidence	Interventional evidence	Clinical readiness
GH/IGF-1 axis	IGF-1R is expressed in retinal vascular, glial, and neuronal compartments. IGF-1R signaling may converge on PI3K/Akt, HIF-1α, NF-κB, AP-1, and VEGF-related pathways. Its effect may be stage-dependent, with potential neurotrophic effects in early disease and angiogenic or leakage-promoting effects in hypoxic or inflamed retina.	Increased ocular IGF-1 can induce diabetes-like retinal vascular changes, including leakage and capillary pathology. Experimental diabetic models suggest that altered IGF-1 signaling can contribute to endothelial dysfunction and retinal vascular injury when insulin signaling is impaired.	Historical observations include regression of severe DR after pituitary infarction, lower retinopathy rates in hypopituitarism or GH-deficient states, DR acceleration during puberty, and retinal disease in acromegaly with glucose dysregulation.	Older somatostatin analog studies suggested possible benefit in selected DR populations but were not biomarker-selected and predated modern anti-VEGF practice. Recent lanreotide data in persistent DME provide early clinical and mechanistic signals but remain preliminary.	Moderate biological plausibility; low-to-moderate clinical readiness. Best suited for biomarker-selected research in pubertal PDR, acromegaly-associated retinal disease, high IGF-1 burden, and persistent or suboptimally responsive DME despite predefined adequate anti-VEGF exposure
HPA–glucocorticoid signaling and related GR/MR balance	Corticosteroid signaling acts through GR and MR pathways. GR signaling in Müller glia may restrain gliosis and support retinal function, whereas MR activation may promote inflammation, leakage, leukostasis, and vascular dysfunction. Cortisol rhythm disturbance may also affect circadian retinal repair.	Experimental work supports a protective role of Müller cell GR signaling under diabetic stress. Aldosterone or MR overactivation can induce inflammatory retinal programs. In rodent models of diabetic and neovascular retinopathy, MR antagonism reduced vascular injury and modified immune-related signals.	Type 2 diabetes, obesity, chronic stress, depression, and sleep disruption are associated with altered HPA activity. Obstructive sleep apnea is linked to DR progression in several clinical datasets, although findings are not fully consistent across studies.	Intravitreal corticosteroids are established for DME but represent local ocular therapy rather than systemic HPA correction. CPAP has shown retinal benefit in selected patients with OSA and NPDR. Finerenone is clinically used for renal and cardiovascular indications, but retinal outcome data remain indirect or preclinical.	Moderate translational readiness. Strongest research settings include OSA-associated DR, recurrent DME, persistent or suboptimally responsive DME despite predefined adequate anti-VEGF exposure, MR-related leakage phenotypes, and patients already receiving MR antagonists for approved systemic indications.
Thyroid hormone axis	Thyroid hormone receptors and local T4-to-T3 conversion through deiodinase activity may regulate retinal metabolism, photoreceptor function, mitochondrial reserve, glial activity, and neurovascular resilience. Low T3 activity may reduce retinal tolerance to hyperglycemic stress.	Retinal development and photoreceptor biology depend on thyroid hormone signaling. Direct DR-specific interventional model evidence remains less developed than for GH/IGF-1 or corticosteroid pathways.	Clinical studies have linked thyroid status, normal-range thyroid hormone variation, lower FT3, resting metabolic rate, systemic inflammation, and DR risk or progression, although evidence is not uniform. Mendelian randomization data are cautious and suggest that thyroid-DR causality remains unresolved.	Treating overt hypothyroidism is standard endocrine care, but there is no established evidence that treating low-normal FT3 prevents DR. Future studies should focus on patients with defined thyroid phenotypes and retinal endpoints.	Low-to-moderate readiness. Best suited for longitudinal cohorts and biomarker studies in low-normal FT3, subclinical hypothyroidism, early NPDR, OCTA progression, and retinal neurodegeneration phenotypes.
Sex steroid axis	Estrogen receptors, androgen-related pathways, and local steroidogenic enzymes are present in retinal and RPE compartments. Estrogenic signaling may support vascular tone, reduce oxidative stress, protect barrier function, and support neural and glial resilience. Androgen excess may interact with insulin resistance and inflammation.	Estradiol has shown protective effects in hyperglycemic Müller glia and endothelial systems, including reduced oxidative stress and improved barrier resistance. Barrier-on-chip and cell-based models support context-dependent sex steroid effects under diabetic stress.	DR may change during puberty, pregnancy, menopause, PCOS, hypogonadism, and other states of altered gonadal hormone signaling. Menopausal status may modify the relationship between systemic metabolic markers and DR or PDR risk.	There is no evidence to support broad sex hormone therapy for DR prevention or treatment. Menopausal hormone therapy has systemic risks and should not be proposed for retinal indications without dedicated evidence.	Low clinical readiness but high phenotyping value. Best suited for registries and prospective cohorts that record menopausal status, pregnancy, PCOS, hormone therapy exposure, androgen markers, lipids, and retinal imaging outcomes.

Akt, protein kinase B; AP-1, activator protein-1; CPAP, continuous positive airway pressure; DME, diabetic macular edema; DR, diabetic retinopathy; FT3, free triiodothyronine; GH, growth hormone; GR, glucocorticoid receptor; HIF-1α, hypoxia-inducible factor-1 alpha; HMB, Hormonal Metabolic Burden; HPA, hypothalamic-pituitary-adrenal; IGF-1, insulin-like growth factor-1; IGF-1R, insulin-like growth factor-1 receptor; MR, mineralocorticoid receptor; NF-κB, nuclear factor kappa B; NPDR, nonproliferative diabetic retinopathy; OCTA, optical coherence tomography angiography; OSA, obstructive sleep apnea; PCOS, polycystic ovary syndrome; PDR, proliferative diabetic retinopathy; PI3K, phosphoinositide 3-kinase; RPE, retinal pigment epithelium; T3, triiodothyronine; T4, thyroxine; VEGF, vascular endothelial growth factor.

## 4 The hormonal metabolic burden hypothesis

### 4.1 Definition, scope, and candidate phenotype-matched research variables

HMB is defined as a multidimensional, axis-specific, time-dependent, and phenotype-matched endocrine-retinal burden profile rather than a cumulative count of abnormal hormone values or a single unvalidated score. In this framework, “burden” refers to the magnitude, duration, timing, co-occurrence, dominant axis, and biological context of endocrine disturbances acting on a diabetic retina that is already shaped by hyperglycemia, blood pressure, kidney disease, lipid status, diabetes duration, and baseline retinopathy severity. Therefore, HMB should not be interpreted as the arithmetic addition of abnormalities in GH/IGF-1 signaling, HPA–glucocorticoid signaling and related GR/MR balance, thyroid hormone signaling, and sex steroid signaling. A predominantly pubertal GH/IGF-1-related profile, sleep apnea with cortisol rhythm disturbance, low-normal FT3 accompanied by neurodegenerative retinal features, and postmenopausal inflammatory or lipid-related acceleration may each represent a biologically distinct endocrine-retinal burden pattern, with different dominant axes, retinal phenotypes, and testable predictions.

For this reason, the current purpose of HMB is not to generate a universal clinical score, a positive/negative laboratory result, or an opaque machine-learning index. It is to provide an axis-specific and phenotype-matched research framework for explaining residual retinal risk and treatment heterogeneity. Within this framework, early or rapidly progressive DR phenotypes are primarily used to test residual-risk and future prevention hypotheses, persistent or suboptimally responsive DME phenotypes are used to test treatment-response heterogeneity, and established proliferative diabetic retinopathy (PDR) remains a setting in which evidence-based ocular treatment takes priority over endocrine-retinal profiling.

At the current stage, HMB is not a panel in the clinical laboratory sense. Its output is a structured research profile comprising conventional DR covariates, a predefined retinal phenotype, a limited set of axis-specific variables selected *a priori*, and longitudinal retinal endpoints. It does not produce a positive/negative test result, a validated high/low burden classification, or an individual treatment recommendation. In future studies, relative burden categories, if used, should be restricted to study-specific analytical strata defined *a priori* according to transparent classification rules. Accordingly, the terms “higher-burden” and “lower-burden” should refer only to comparative research strata within a given protocol and should not be interpreted as validated clinical classifications, diagnostic labels, treatment thresholds, or guides to individual treatment. Within this framework, candidate phenotype-matched research variables could be developed in stages. At the first stage, these variables may include accessible clinical research measures, such as age-adjusted IGF-1, FT3 or thyroid category, sleep apnea status, cortisol rhythm when available, menopausal or sex steroid context, kidney disease, and the current retinal phenotype. At the second stage, mechanistic research markers could be added, including insulin-like growth factor-binding protein 3 (IGFBP-3), inflammatory proteins, MR pathway markers, methylation signatures, and quantitative retinal imaging features. At a later stage, a composite HMB index could be explored only if longitudinal data identify reproducible variables, appropriate weighting, interaction terms, phenotype specificity, and incremental predictive value beyond standard DR models. Until then, HMB should remain a transparent multidimensional research profile, with the dominant axis, level of evidence, matched retinal phenotype, and longitudinal endpoint clearly reported.

HMB also encourages better timing in research design. In established proliferative DR, evidence-based ocular treatment remains the priority. Endocrine assessment should not delay evidence-based ocular treatment. The potential value of HMB may be greatest earlier in the disease course, when patients show accelerated neurodegeneration, early ischemia, recurrent edema, or suboptimal treatment response before irreversible structural complications have developed. In these settings, endocrine-related risk may still be measurable and potentially modifiable. Timing also matters because endocrine-related interventions are unlikely to act as rapidly as intravitreal therapy. CPAP adherence, thyroid correction, weight change, or MR blockade may require months to influence retinal tissue. For this reason, research protocols should align endpoints and observation windows with the expected biology of the axis being studied. Very short endpoints may be insufficient for evaluating endocrine-retinal modification, whereas illustrative longitudinal observation windows of 12–24 months may be considered in studies assessing OCTA change, injection burden, edema recurrence, or retinal neurodegeneration. These variables, sampling approaches, and follow-up windows are research-design considerations and should not be interpreted as recommendations for routine clinical testing or follow-up. The HMB framework proposes that several mild endocrine disturbances may converge with hyperglycemia on shared retinal injury pathways, thereby increasing residual retinal risk. The interaction architecture underlying this concept is developed below.

### 4.2 Shared downstream pathways: oxidative stress, inflammation, VEGF, and neurodegeneration

Oxidative stress is a central pathway in DR. Hyperglycemia increases mitochondrial stress, advanced glycation end products, polyol pathway activity, protein kinase C signaling, and nicotinamide adenine dinucleotide phosphate (NADPH) oxidase activity. A 2020 review summarized the role of oxidative stress as both a driver and amplifier of retinal injury (48). HMB adds that endocrine axes may change oxidative tone. IGF-1 signaling can change mitochondrial and growth-factor pathways. Cortisol and MR activation may increase inflammatory oxidative stress. Low T3 may reduce mitochondrial efficiency. Estrogen withdrawal may remove antioxidant support. Inflammation is the second shared pathway. DR is no longer viewed as a purely microvascular disease. It includes glial activation, cytokine release, leukostasis, microglial response, and barrier cell dysfunction. A 2023 review emphasized inflammation as a link between neural and vascular injury in the diabetic retina (49). HMB fits this model. Each axis can shape inflammatory threshold. GR loss in Müller cells, MR activation, low T3, and estrogen withdrawal may all move the retina toward a more reactive state.

VEGF remains the most important final common pathway for leakage and neovascularization. Yet persistent edema despite frequent intravitreal therapy reminds us that VEGF is not the whole disease. Some patients have persistent fluid despite repeated treatment, whereas others have ischemia, inflammation, or neurodegeneration that cannot be reduced to VEGF alone. HMB may help identify upstream reasons for this heterogeneity. A patient with persistent DME and an endocrine-retinal profile characterized by greater magnitude, persistence, or co-occurrence of prespecified axis-specific disturbances may warrant a different research pathway from a patient whose edema responds quickly to VEGF blockade. Neurodegeneration is the fourth pathway. Inner retinal thinning, ganglion cell dysfunction, reduced contrast sensitivity, and electroretinographic changes can occur early in diabetes. This matters because endocrine signals often act strongly on neural and glial cells. Low IGF-1 survival signaling, GR loss, low T3 effect, and estrogen withdrawal may each reduce retinal reserve before obvious vascular lesions appear. HMB therefore should be tested with OCT and functional endpoints, not only fundus photographs.

### 4.3 Cross-axis hormonal interactions: additive, synergistic, hierarchical, and sequential patterns

A central premise of HMB is that endocrine axes may interact with each other and with the diabetic retinal environment. However, HMB does not assume universal synergy. Depending on retinal stage, cellular compartment, systemic metabolic background, and clinical context, multi-axis effects may be additive, supra-additive, hierarchical, antagonistic, or temporally sequential. The interaction structure is therefore not a settled assumption but a key testable component of the HMB hypothesis.

The first layer of the interaction model is the sensitized diabetic retinal substrate. Hyperglycemia, hemodynamic stress, diabetes duration, kidney disease, dyslipidemia, ischemia, baseline retinopathy severity, and prior metabolic memory may lower the threshold for retinal injury. These conventional factors define the background in which endocrine signals acquire retinal meaning. The same endocrine profile may have limited relevance in a low-risk retina but greater impact in a retina already exposed to chronic hyperglycemia, inflammation, ischemia, or barrier dysfunction.

The second layer consists of axis-specific main effects. GH/IGF-1 signaling may shift between neurotrophic and angiogenic effects depending on retinal stage and insulin signaling. HPA–glucocorticoid signaling and related GR/MR balance may influence systemic insulin resistance, Müller cell resilience, inflammation, vascular leakage, and edema-related phenotypes. Thyroid hormone signaling may affect mitochondrial function, local T3 availability, and retinal metabolic reserve. Sex steroid signaling may modify oxidative stress, lipid-inflammatory interactions, vascular tone, and blood-retinal barrier stability. These axis-specific effects are biologically meaningful, but HMB proposes that their retinal effect is often context-dependent rather than linear.

The third layer is conditional cross-axis interaction. In an additive pattern, several mild endocrine disturbances may each reduce retinal reserve modestly, but together push the retina beyond a threshold for edema, neurodegeneration, or vascular progression. In a supra-additive or synergistic pattern, one axis may change the downstream signaling environment of another. For example, cortisol-related insulin resistance may redirect IGF-1 signaling toward angiogenic or leakage pathways; estrogen withdrawal may reduce anti-inflammatory or barrier support while MR-related inflammation increases; and low T3-related metabolic vulnerability may amplify the effect of reduced neurotrophic signaling. In a hierarchical pattern, one axis may predominate under a specific clinical condition. GH/IGF-1 may be more relevant in pubertal or young-onset proliferative phenotypes; sleep apnea, cortisol rhythm disturbance, and MR-related mechanisms may be more relevant in recurrent or persistent edema; low-normal FT3 may be more relevant in early neurodegenerative or low-reserve phenotypes; and menopausal transition may modify lipid-inflammatory and vascular susceptibility. These examples should be interpreted as hypotheses for enrichment and testing, not as established clinical categories.

The fourth layer is temporal organization. HMB may operate through a priming–trigger–maintenance pattern. During priming, long-standing hyperglycemia together with mild endocrine imbalance may reduce retinal metabolic, glial, vascular, or barrier reserve. During triggering, a transition such as puberty, pregnancy, menopause, sleep-related hypoxia, chronic stress, rapid glycemic improvement, medication change, or worsening renal-metabolic status may accelerate retinal change. During maintenance, oxidative stress, inflammation, Müller gliosis, blood-retinal barrier breakdown, and epigenetic metabolic memory may sustain vulnerability even after one endocrine marker improves. This temporal structure links cross-axis interaction with hormonal metabolic memory and explains why single time-point hormone measurement may be insufficient.

**Figure 3:**
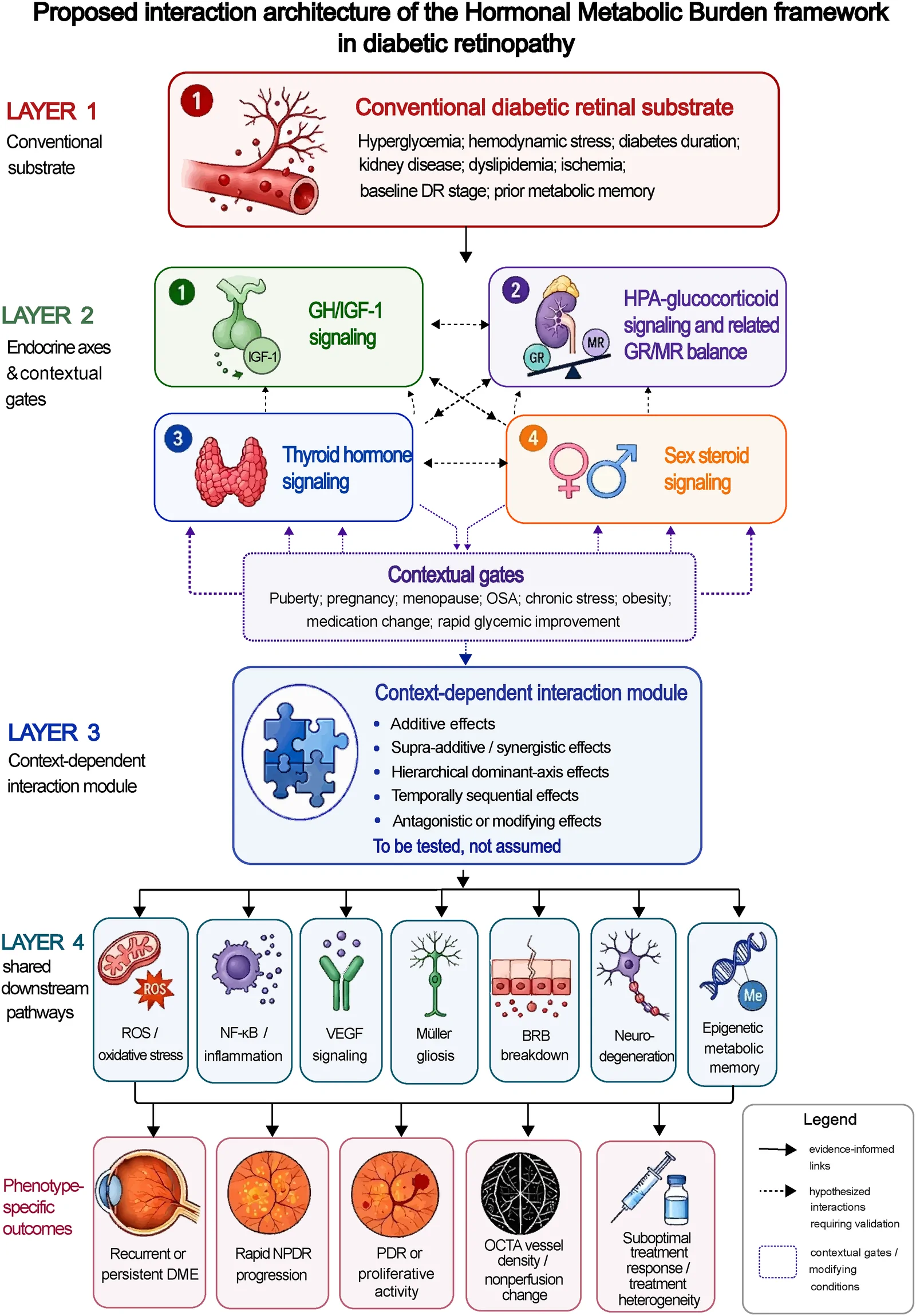
Proposed interaction architecture of the hormonal metabolic burden framework in diabetic retinopathy. The HMB framework proposes that endocrine-retinal risk arises from interactions between a sensitized diabetic retinal substrate, selected endocrine axes, contextual gates, and shared retinal injury pathways. Conventional diabetic factors, including hyperglycemia, hemodynamic stress, diabetes duration, kidney disease, dyslipidemia, ischemia, baseline retinopathy severity, and prior metabolic memory, form the background substrate rather than being replaced by HMB. The initial HMB core includes GH/IGF-1 signaling, HPA–glucocorticoid signaling and related GR/MR balance, thyroid hormone signaling, and sex steroid signaling. Clinical and physiological states such as puberty, pregnancy, menopause, obstructive sleep apnea, chronic stress, obesity, medication change, and rapid glycemic improvement may function as contextual gates that modify which axis becomes dominant. Multi-axis effects may be additive, supra-additive/synergistic, hierarchical, antagonistic, or temporally sequential; these interaction patterns are proposed as testable hypotheses rather than established causal pathways. Downstream convergence may involve ROS-driven oxidative stress, NF-κB-mediated inflammation, VEGF signaling, Müller gliosis, blood-retinal barrier breakdown, neurodegeneration, and epigenetic metabolic memory. These pathways may contribute to recurrent or persistent DME, rapid NPDR progression, proliferative activity, OCT/OCTA progression, and treatment heterogeneity. Solid arrows indicate evidence-supported substrate-to-pathway or pathway-to-phenotype links. Dashed arrows indicate hypothesized cross-axis HMB interactions that require validation. Dotted borders indicate contextual gates or modifying conditions. BRB, blood-retinal barrier; DME, diabetic macular edema; DR, diabetic retinopathy; GH, growth hormone; GR, glucocorticoid receptor; HMB, Hormonal Metabolic Burden; HPA, hypothalamic–pituitary–adrenal; IGF-1, insulin-like growth factor-1; Me, methyl group; MR, mineralocorticoid receptor; NF-κB, nuclear factor kappa B; NPDR, nonproliferative diabetic retinopathy; OCT, optical coherence tomography; OCTA, optical coherence tomography angiography; OSA, obstructive sleep apnea; PDR, proliferative diabetic retinopathy; ROS, reactive oxygen species; VEGF, vascular endothelial growth factor.

This interaction model changes how HMB should be tested. The key question is not whether one hormone is universally associated with DR, but whether a predefined endocrine-retinal profile improves prediction or mechanistic explanation in a matched phenotype. Clinical cohorts should therefore test interaction terms, dominant-axis patterns, time-varying endocrine measures, and retinal endpoints selected according to the presumed mechanism. Experimental systems should use factorial designs to determine whether combined endocrine and glucose stress produces additive, supra-additive, antagonistic, or sequential effects on oxidative stress, inflammatory signaling, VEGF expression, glial activation, barrier resistance, apoptosis, or epigenetic marks. This proposed interaction architecture is summarized in **Figure 3**.

### 4.4 Epigenetic hormonal metabolic memory

The maintenance phase of the proposed interaction model is closely related to metabolic memory. Metabolic memory is one of the strongest reasons to look beyond current HbA1c. In DR, prior hyperglycemia can leave lasting changes in oxidative stress and inflammatory pathways. Epigenetic changes at antioxidant genes such as superoxide dismutase 2 (SOD2) and nuclear factor erythroid 2–related factor 2 (NRF2) pathways have been linked to persistent retinal damage (50, 51). This helps explain why retinal disease can progress even after later glycemic improvement.

HMB extends this idea. Hormones are transcriptional signals. Glucocorticoids bind nuclear receptors and alter gene expression. Thyroid hormone regulates metabolic and mitochondrial transcription. Estrogen changes chromatin and inflammatory responses. IGF-1 alters growth and survival pathways that can affect epigenetic enzymes. It is therefore plausible that long-standing hormonal stress may leave a retinal memory, especially when it occurs with hyperglycemia.

Hormonal metabolic memory remains a hypothesis and should not be presented as proven. However, it generates several testable predictions. Participants meeting prespecified criteria for a study-specific higher-burden endocrine-retinal research stratum are hypothesized to show persistent retinal risk even after improvement in one endocrine marker. Earlier correction of a matched endocrine disturbance is hypothesized to have a greater retinal effect than later correction. Organoid and barrier-on-chip models should show durable epigenetic marks after combined endocrine and glucose stress. These predictions can be tested in longitudinal cohorts and factorial retinal models that evaluate not only single-axis effects but also combined, timed, and persistent endocrine-glucose stress.

### 4.5 Testable predictions

The HMB hypothesis is falsifiable, which strengthens its scientific value. First, a predefined multidimensional HMB profile should predict DR progression beyond HbA1c, blood pressure, diabetes duration, kidney function, lipids, and baseline retinopathy grade. Second, participants assigned *a priori* to a study-specific higher-burden endocrine-retinal research stratum should show faster OCT or OCTA change than participants in a corresponding lower-burden stratum with similar standard risk profiles, particularly with respect to inner retinal thinning, vessel density loss, nonperfusion, or persistent edema. Third, patients with persistent or suboptimally responsive DME despite an adequate anti-VEGF treatment course should show a different endocrine-retinal profile from good responders. Fourth, later-stage biomarker-selected endocrine modification should improve retinal endpoints more than the same intervention in unselected patients.

A negative study would also be useful. If a well-designed cohort measures endocrine axes repeatedly and HMB adds no predictive value, the hypothesis weakens. If an endocrine intervention changes the biomarker but not the retina in a correctly selected group, the causal claim weakens. If organoids or barrier-on-chip models show no reproducible interaction pattern, no phenotype specificity, and no effect beyond single-axis exposure, the interaction component of HMB would be weakened. If only additive effects are observed, the synergy component would be weakened, but the broader multi-axis burden concept may still remain testable. HMB should be built with this discipline.

The most important statistical test is incremental value. HMB must improve prediction beyond models that already include age, diabetes duration, HbA1c, blood pressure, kidney function, and baseline retinopathy. A biomarker that is associated with DR but adds no clinical information may still be biologically interesting, but it will not change care. The bar should be high. HMB should improve discrimination, calibration, risk reclassification, treatment-response stratification, or mechanistic trial enrichment. Otherwise, it should remain a research concept (**Table 3**).

**Table 3:** Testable predictions of the HMB hypothesis.

Prediction	Required study design	Biomarker	Retinal endpoint	Expected implication
A prespecified multidimensional HMB research profile provides incremental prediction of DR progression beyond standard risk factors.	Prospective longitudinal endocrine-retinal cohort with adjustment for age, diabetes duration, HbA1c, blood pressure, kidney function, lipid status, and baseline DR grade.	Age-adjusted IGF-1, FT3 or thyroid category, cortisol rhythm, sleep apnea status, menopausal or sex steroid context, kidney markers, and standard metabolic variables.	ETDRS step progression, incident DME, PDR development, OCT/OCTA progression, vessel density loss, nonperfusion, and inner retinal thinning.	If positive, HMB would provide incremental predictive value beyond standard DR risk models. If negative, the hypothesis would remain biologically interesting but clinically less useful.
Participants assigned a priori to a study-specific higher-burden endocrine-retinal research stratum are hypothesized to show faster OCT/OCTA progression than participants in a corresponding lower-burden stratum with similar standard risk profiles.	Matched cohort or longitudinal imaging study using prespecified, transparent, and study-specific burden strata, with adjustment for glycemic control, blood pressure, renal status, and baseline retinopathy severity.	Repeated multi-axis endocrine-retinal measurements used to define the study-specific research strata; candidate axis-specific variables may include IGF-1 SDS, FT3, cortisol rhythm, OSA severity, and sex steroid context.	Ganglion cell–inner plexiform layer thinning, inner retinal thinning, vessel density loss, FAZ enlargement, capillary nonperfusion, edema recurrence, and DME morphology.	Supports the hypothesis that relative endocrine-retinal burden modifies retinal vulnerability and may identify a higher-risk imaging phenotype. These strata would remain study-specific analytical categories rather than validated clinical classifications.
Persistent or suboptimally responsive DME despite predefined adequate anti-VEGF exposure is enriched for selected endocrine signals.	Nested responder/non-responder study within a treated DME cohort using standardized anti-VEGF exposure and predefined response criteria.	IGF-1 SDS, OSA status, nocturnal oxygen desaturation, cortisol rhythm, MR-related risk profile, albuminuria, renal function, FT3, and inflammatory markers.	Persistent intraretinal or subretinal fluid, central macular thickness, edema volume, recurrence interval, injection burden, and need for treatment switch.	If endocrine signals are enriched in non-responders, HMB may help define adjunctive trial populations and explain treatment heterogeneity.
Combined glucose and endocrine stress causes greater retinal injury than single-axis exposure.	Factorial experiments using human retinal organoids, Müller glia-endothelial systems, RPE models, or inner blood-retinal barrier-on-chip platforms.	Controlled high glucose exposure combined with IGF-1 alteration, cortisol or aldosterone/MR activation, low T3 conditions, estradiol withdrawal, or androgen-related stress.	ROS generation, NF-κB activation, VEGF expression, cytokine release, Müller gliosis, endothelial permeability, barrier resistance, apoptosis, and epigenetic marks.	Demonstrates whether HMB is additive or synergistic at the tissue-model level and helps prioritize axes for clinical testing.
Biomarker-selected endocrine correction improves retinal endpoints more than unselected intervention.	Biomarker-selected enrichment trial, pragmatic registry study, or embedded clinical trial in participants selected by both retinal phenotype and endocrine profile.	Improvement in the targeted endocrine marker, such as CPAP adherence and oxygen desaturation improvement, thyroid correction, MR pathway modulation, or IGF-1 pathway modulation.	OCT/OCTA change, DME recurrence, treatment burden, ETDRS step progression, vessel density, nonperfusion, and retinal neurodegeneration markers.	Supports precision endocrine-retinal intervention only if retinal benefit is greater in biomarker-selected patients than in unselected patients.
Earlier endocrine correction has a greater retinal effect than late correction if hormonal metabolic memory exists.	Longitudinal cohort or early-versus-late intervention study with repeated endocrine measurements and long-term retinal imaging follow-up.	Duration and severity of endocrine disturbance, time to correction, HbA1c trajectory, cortisol rhythm, FT3, IGF-1 status, sex steroid context, and epigenetic or inflammatory markers.	Long-term OCT/OCTA progression, neurodegeneration, edema recurrence, injection burden, and delayed DR progression after endocrine stabilization.	If early correction has greater retinal benefit than late correction, this would support the concept of hormonal metabolic memory and justify earlier phenotype-based research.

CPAP, continuous positive airway pressure; DME, diabetic macular edema; DR, diabetic retinopathy; ETDRS, Early Treatment Diabetic Retinopathy Study; FAZ, foveal avascular zone; FT3, free triiodothyronine; HbA1c, glycated hemoglobin; HMB, Hormonal Metabolic Burden; IGF-1, insulin-like growth factor-1; MR, mineralocorticoid receptor; NF-κB, nuclear factor kappa B; OCT, optical coherence tomography; OCTA, optical coherence tomography angiography; OSA, obstructive sleep apnea; PDR, proliferative diabetic retinopathy; ROS, reactive oxygen species; RPE, retinal pigment epithelium; SDS, standard deviation score; T3, triiodothyronine; VEGF, vascular endothelial growth factor.

## 5 A research agenda for endocrine-retinal phenotyping

### 5.1 Candidate phenotypes for HMB research enrichment

The initial use of HMB should be research enrichment rather than clinical screening. Its intended entry point is an atypical or disproportionate retinal course, not routine endocrine stratification of all patients with DR. One phenotype is early or rapid NPDR. This includes patients whose retinal disease appears too soon or progresses too quickly for their diabetes duration, HbA1c, blood pressure, and kidney status. A second phenotype is persistent DME with suboptimal anti-VEGF response, preferably defined in research protocols as center-involving DME that persists despite an adequate treatment course. Persistent center-involving edema after repeated injections may reflect inflammation, MR activation, sleep apnea, kidney disease, or other systemic drivers. A third phenotype is pubertal or young-onset proliferative DR. GH/IGF-1 biology may be more active here. A fourth phenotype is postmenopausal acceleration. Sex steroid change, lipid change, and inflammation may interact. A fifth phenotype is DR with thyroid dysfunction or low-normal FT3. A sixth phenotype is DR with untreated sleep apnea. The goal is not to label these patients as “hormonal DR,” which would overstate the current evidence. Rather, the goal is to identify retinal phenotypes in which endocrine-retinal study is biologically rational. These phenotypes should be used to define research cohorts or mechanistic subgroups, not to trigger routine endocrine testing in standard retinal practice. Each phenotype should lead to a predefined, focused research variable set rather than an open-ended endocrine test list (**Table 4**).

**Table 4:** Retinal phenotypes for research-oriented HMB enrichment.

Retinal phenotype	Why HMB is plausible	Focused endocrine-retinal research variables	Suggested study design	Key caution
Rapid or early NPDR	Retinal disease appears earlier or progresses faster than expected based on diabetes duration, HbA1c, blood pressure, kidney disease, and baseline DR grade. Endocrine stress may reduce retinal reserve and amplify standard metabolic injury.	HbA1c trend, recent HbA1c reduction, blood pressure, kidney function, albuminuria, lipids, IGF-1 SDS, TSH/FT4/FT3, sleep apnea screen, cortisol rhythm if available, OCT/OCTA baseline phenotype.	Residual-risk longitudinal cohort with standard risk adjustment and repeated retinal imaging.	Exclude common explanations first, including unrecognized poor glycemic history, hypertension, renal disease, poor screening history, and baseline grading error.
Persistent or suboptimally responsive DME despite predefined adequate anti-VEGF exposure	Persistent edema despite adequate anti-VEGF exposure may reflect non-VEGF drivers such as inflammation, MR activation, sleep apnea, renal disease, cortisol rhythm disturbance, or altered GH/IGF-1 signaling.	Standardized anti-VEGF treatment history, OCT edema morphology, IGF-1 SDS, OSA screen or polysomnography, nocturnal oxygen desaturation, cortisol rhythm, albuminuria, renal function, MR-related risk, FT3, inflammatory markers.	Nested responder/non-responder study or biomarker-enriched adjunctive trial.	Confirm adequate anti-VEGF exposure and exclude tractional components, ischemic maculopathy, poor injection adherence, undertreatment, and irreversible structural damage.
Pubertal or young-onset PDR	Puberty combines GH/IGF-1 activation, sex steroid change, insulin resistance, metabolic instability, and sometimes rapid glycemic correction. These factors may converge on VEGF-related and angiogenic pathways.	Pubertal status, age-adjusted IGF-1, diabetes type and duration, HbA1c trend, rate of HbA1c reduction, insulin regimen change, OCT/OCTA ischemic markers, neovascular activity, renal status.	Prospective adolescent or young-onset diabetes cohort; mechanistic substudy in patients with rapid angiogenic progression.	Endocrine research must not delay urgent anti-VEGF, laser, or surgical treatment when PDR is active.
Postmenopausal acceleration	Menopause may reduce estrogenic vascular and neural support and may interact with lipid change, inflammation, body composition, insulin resistance, and thyroid status.	Menopausal status, age at menopause, hormone therapy exposure, estradiol context, SHBG, androgen markers if relevant, lipids, HbA1c trend, TSH/FT4/FT3, OCT/OCTA progression, DME history.	Registry-based cohort or prospective postmenopausal DR imaging cohort with age- and duration-matched controls.	Do not infer that estrogen therapy is indicated for DR. Age, diabetes duration, cardiovascular risk, lipid status, and renal disease are major confounders.
DR with thyroid dysfunction or low-normal FT3	Thyroid hormone signaling may influence retinal mitochondrial reserve, glial activity, neurodegeneration, and pericyte vulnerability. Low-normal FT3 may mark a low-reserve metabolic and inflammatory phenotype.	TSH, FT4, FT3, thyroid antibody status if clinically indicated, resting metabolic or inflammatory markers if available, renal function, albuminuria, OCT/OCTA neurodegeneration and vessel density endpoints.	Longitudinal thyroid-retina cohort; subgroup analysis of patients with overt thyroid disease, subclinical hypothyroidism, or low-normal FT3.	Treat thyroid disease according to endocrine indications. Low-normal FT3 alone should not be treated as a retinal therapeutic target without trial evidence.
DR with untreated OSA or chronic sleep disruption	OSA can produce intermittent hypoxia, sympathetic activation, sleep fragmentation, cortisol rhythm disturbance, inflammation, and vascular stress, all of which may aggravate retinal leakage or progression.	STOP-BANG or Epworth screening, polysomnography when available, apnea-hypopnea index, oxygen desaturation index, CPAP adherence, cortisol rhythm, blood pressure, BMI, renal function, OCT/OCTA endpoints.	Prospective OSA-DR cohort or CPAP adherence study with retinal imaging endpoints.	Obesity, hypertension, renal disease, and poor metabolic control can confound the sleep-retina relationship. Objective adherence data are essential.
Early retinal neurodegeneration disproportionate to vascular lesions	Inner retinal thinning, ganglion cell dysfunction, or early functional loss may reflect reduced neurovascular reserve before severe vascular signs appear. Endocrine effects on IGF-1 signaling, thyroid hormone, GR activity, and sex steroid status may be relevant.	OCT ganglion cell-inner plexiform layer thickness, inner retinal thickness, OCTA vessel density, visual function measures if available, IGF-1 SDS, FT3, cortisol rhythm, sleep status, sex steroid context, medication history.	Imaging-based neurodegeneration cohort; mechanistic organoid or retinal cell model study matched to clinical phenotype.	Exclude glaucoma, optic neuropathy, high myopia, media opacity, segmentation error, and non-diabetic neuroretinal disease before attributing changes to HMB.

### 5.2 Proposed phenotype-guided workflow for research cohort enrichment

At present, no validated HMB score, axis-specific retinal cutoff, screening threshold, or evidence-based endocrine intervention pathway exists for DR. HMB profiling should therefore be restricted to ethically approved research protocols and should not alter or delay established ocular or systemic care. The proposed workflow is intended for cohort enrichment, prospective data collection, and hypothesis testing rather than for immediate clinical decision-making.

The first step in a research cohort is conventional DR risk characterization. This includes diabetes type and duration, HbA1c and its recent trajectory, blood pressure, kidney function and albuminuria, lipid status, pregnancy status when relevant, medication changes, baseline retinopathy grade, OCT findings, OCTA findings when available, and treatment response. These variables are required because HMB can only be evaluated meaningfully after established DR risk factors have been measured and modeled, consistent with existing DR risk prediction and risk-based screening models (52–54).

The second step is phenotype-based research stratification. Participants with a retinal course that is proportionate to their standard risk profile may serve as a reference or comparator group within the cohort. Participants with atypical or disproportionate retinal phenotypes may be enriched for phenotype-matched endocrine-retinal research measurements. For pubertal or young-onset proliferative patterns, candidate variables may include age-adjusted IGF-1 and markers of GH/IGF-1 bioactivity. For recurrent or persistent edema, sleep-related risk, or suspected stress-related physiology, candidate variables may include objective sleep apnea metrics, oxygen desaturation, CPAP adherence where applicable, cortisol rhythm research measures, kidney disease, blood pressure, potassium, and MR-related medication exposure. For thyroid-linked phenotypes, candidate variables may include TSH, FT4, FT3, thyroid category, and inflammatory or metabolic covariates. For postmenopausal or sex steroid-linked acceleration, candidate variables may include menopausal status, hormone therapy exposure, sex steroid context, SHBG or androgen markers where appropriate, lipids, and age-related vascular risk. These variables should be collected under predefined protocols and interpreted as research biomarkers rather than clinical retinal decision tools.

**Figure 4:**
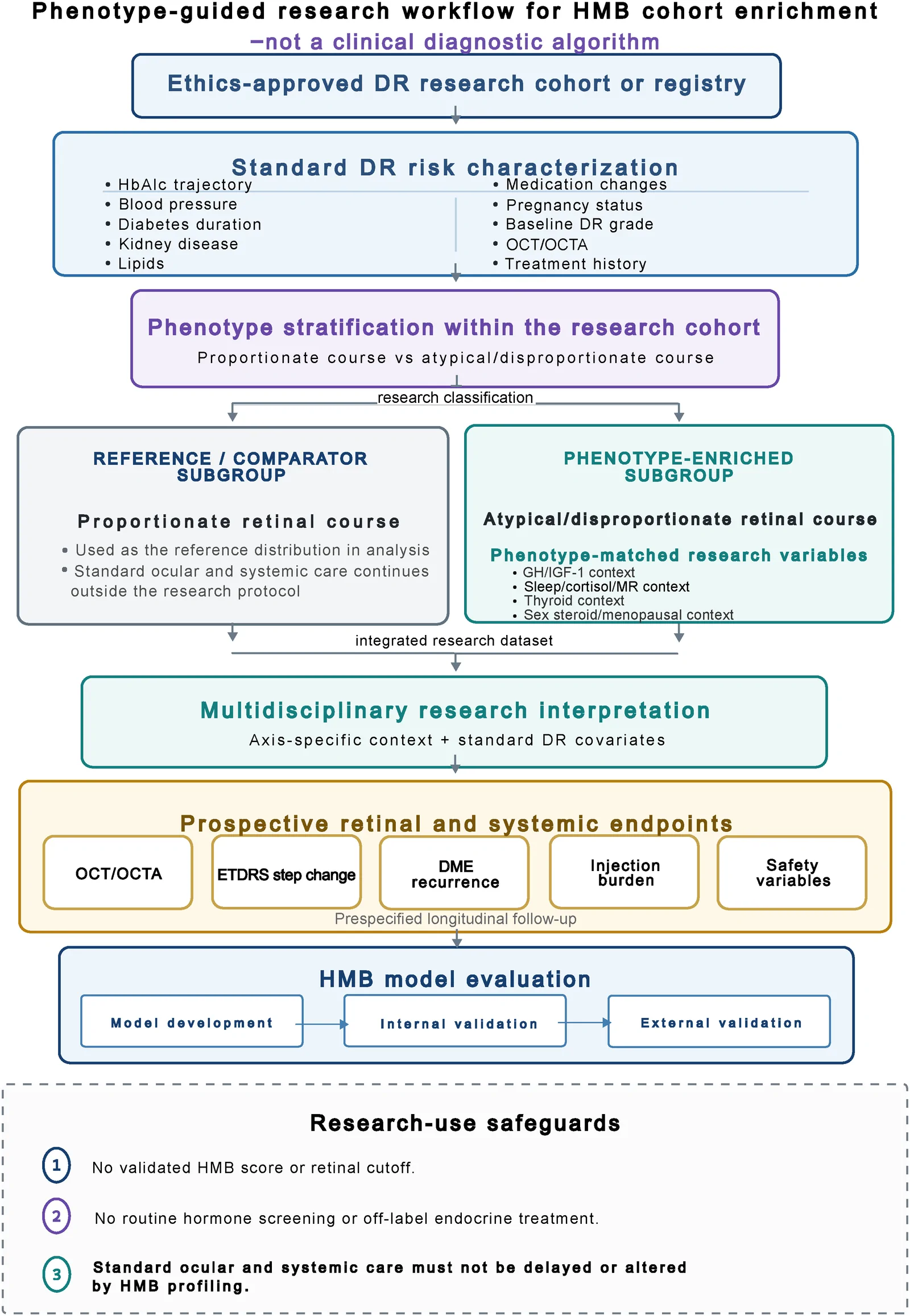
Phenotype-guided research workflow for HMB cohort enrichment—not a clinical diagnostic algorithm. This workflow illustrates how the HMB framework could be tested in ethically approved DR research cohorts or registries. All participants should first undergo standard DR risk characterization, including HbA1c trajectory, blood pressure, diabetes duration, kidney disease, lipid status, medication changes, pregnancy status when relevant, baseline DR grade, OCT/OCTA findings, and treatment history. Phenotype stratification is then performed within the research cohort. Participants with retinal findings proportionate to their standard risk profile may serve as a reference or comparator subgroup, while those with atypical or disproportionate retinal phenotypes may undergo phenotype-matched research variable collection. Candidate variables may include GH/IGF-1 context, sleep/cortisol/MR context, thyroid context, and sex steroid or menopausal context, depending on the predefined phenotype. Multidisciplinary research interpretation should integrate axis-specific measurements with standard DR covariates. The output of this workflow is prospective data collection, retinal endpoint selection, model development, internal validation, and external validation, not immediate clinical decision-making. No validated HMB score, retinal cutoff, or endocrine intervention pathway currently exists for DR; therefore, HMB profiling should not be used for routine hormone screening, off-label endocrine treatment, or any delay or alteration of established ocular and systemic care. Arrows indicate proposed research workflow steps and do not represent causal biological links or clinical decision rules. DME, diabetic macular edema; DR, diabetic retinopathy; ETDRS, Early Treatment Diabetic Retinopathy Study; GH, growth hormone; HbA1c, glycated hemoglobin; HMB, Hormonal Metabolic Burden; IGF-1, insulin-like growth factor-1; MR, mineralocorticoid receptor; OCT, optical coherence tomography; OCTA, optical coherence tomography angiography.

Future studies may benefit from multidisciplinary interpretation by retinal and endocrine investigators. The retinal phenotype should define the research question, endocrine expertise may support contextual interpretation of axis-specific measurements, and the protocol should prospectively define retinal and systemic endpoints. A focused phenotype-matched research variable set is preferable to broad, open-ended endocrine testing. The key research questions should be prespecified: What is the atypical retinal feature? Which endocrine axis is biologically plausible? Which measurement changes the research classification? What retinal endpoint will be followed? What standard clinical variables must be adjusted for? This structure is intended to reduce overtesting and overinterpretation while allowing HMB to be tested as a disciplined research framework (**Figure 4**).

### 5.3 Boundaries before interventional translation

Interventional translation should be regarded as a later-stage research objective rather than an immediate clinical implication of HMB. At the current stage, endocrine modification cannot be recommended for the prevention or treatment of DR on the basis of HMB profiling. Treating established endocrine or systemic disease, such as biochemical thyroid disease, obstructive sleep apnea, kidney disease, hypertension, dyslipidemia, or approved renal-cardiovascular indications for MR antagonism, remains part of standard systemic care. However, using endocrine interventions specifically to improve retinal outcomes requires retinal endpoint evidence.

Before biomarker-selected interventional studies are justified, several prerequisites should be met. First, the endocrine-retinal phenotype should be reproducible in longitudinal cohorts. Second, the candidate HMB profile should improve prediction beyond standard DR risk variables. Third, mechanistic models should support a plausible retinal pathway. Fourth, the proposed intervention should have a clear systemic safety framework and preferably an accepted non-ocular indication. Only after these criteria are met should interventional studies test whether modifying a matched endocrine axis changes predefined retinal endpoints. These endpoints should not rely only on visual acuity, because visual acuity may be too late and variable for early endocrine-retinal studies. OCT-based retinal structural abnormalities can reflect long-term metabolic exposure, as shown in the Diabetes Control and Complications Trial/Epidemiology of Diabetes Interventions and Complications (DCCT/EDIC) OCT study (55). Candidate endpoints may therefore include OCT/OCTA progression, edema recurrence, injection burden, Early Treatment Diabetic Retinopathy Study (ETDRS) step change, inner retinal thickness, ganglion cell-inner plexiform layer measures, vessel density, foveal avascular zone size, nonperfusion, and neurodegenerative measures. Accordingly, early HMB research should prioritize phenotyping, longitudinal prediction, and mechanistic validation. Interventional examples, such as retinal endpoint studies in patients already receiving MR antagonists for approved indications, CPAP studies with objective adherence data, thyroid studies in patients with defined thyroid phenotypes, or carefully selected GH/IGF-1-related trials, should be interpreted as later-stage hypotheses. They should not be presented as current retinal treatment strategies.

## 6 Prioritized validation roadmap and go/no-go criteria

HMB validation should proceed in phases rather than as a parallel list of all possible methods. The first priority is to determine whether HMB can be measured reproducibly and whether it adds longitudinal predictive value beyond established DR risk variables. Mechanistic models, genetic tools, and machine learning should be used only after the core variables, retinal phenotypes, and repeated-measurement strategy have been defined. Biomarker-selected interventional studies should be considered only after clinical reproducibility and mechanistic plausibility have been established. A staged validation pathway with advancement criteria is summarized in **Table 5**.

**Table 5:** Prioritized validation roadmap and go/no-go criteria for the Hormonal Metabolic Burden framework.

Phase	Main question	Preferred design	Go/no-go advancement criterion
Phase 1: essential near-term validation	Can HMB be measured reproducibly, and does it add predictive value beyond standard DR risk factors?	Prospective repeated-measures endocrine-retinal cohort with predefined core axes, contextual variables, OCT/OCTA phenotypes, and standard DR covariates	Advance only if axis-specific measures are reproducible and HMB improves discrimination, calibration, risk reclassification, phenotype prediction, or treatment-response stratification beyond standard variables
Phase 2: external and mechanistic validation	Are HMB phenotypes, axis-specific patterns, and interaction structures externally reproducible and biologically plausible?	Independent cohorts with predefined interaction analyses; factorial human retinal organoid and blood-retinal barrier-on-chip experiments; auxiliary Mendelian randomization, pharmacogenomic, or omics analyses for target prioritization	Advance only if effect direction is consistent across cohorts and mechanistic systems support plausible additive, supra-additive, antagonistic, or temporally persistent endocrine-glucose interactions
Phase 3: long-term interventional validation	Does modifying a matched endocrine axis improve predefined retinal outcomes in a selected phenotype?	Biomarker-selected trials or pragmatic retinal endpoint studies in patients with matched phenotypes and accepted systemic indications where possible	Advance toward clinical translation only if retinal benefit is demonstrated with acceptable systemic safety; stop or revise if biomarkers change without retinal benefit or if safety is unacceptable

DR, diabetic retinopathy; HbA1c, glycated hemoglobin; HMB, Hormonal Metabolic Burden; OCT, optical coherence tomography; OCTA, optical coherence tomography angiography. Note: Standard DR variables include age, diabetes duration, HbA1c and its trajectory, blood pressure, kidney function, lipid status, treatment history, and baseline retinopathy severity. HMB should not proceed to broad scoring, routine screening, or interventional testing unless the preceding phase meets its advancement criterion.

### 6.1 Phase 1: prospective endocrine-retinal cohorts and incremental prediction

The essential first phase is a prospective repeated-measures endocrine-retinal cohort. Its purpose is not to test treatment, but to determine whether HMB can be measured reproducibly and whether it improves longitudinal prediction beyond standard DR risk factors. The cohort should predefine the four core axes, contextual gates, conventional DR variables, retinal phenotypes, sampling schedule, assay methods, and statistical analysis plan. For an illustrative Phase 1 prospective cohort, the candidate HMB research variable set could include IGF-1, TSH, FT4, FT3, morning and late-night salivary cortisol when feasible, sex steroid indices when appropriate, sleep apnea status, HbA1c and its trajectory, blood pressure, kidney function, lipids, medication changes, treatment history, and retinal imaging.

Repeated measurement is essential because hormone levels vary by age, sex, sleep, drugs, kidney disease, obesity, menstrual status, acute illness, and systemic inflammation. Single time-point testing may identify associations but cannot define stable endocrine-retinal burden. The main Phase 1 question should be: does a predefined HMB profile improve prediction of DR progression, OCT/OCTA change, recurrent edema, injection burden, or neurodegenerative measures beyond age, diabetes duration, HbA1c, blood pressure, kidney function, lipids, and baseline retinopathy severity? Advancement beyond Phase 1 should require reproducible axis-specific measurements and measurable incremental value, such as improved discrimination, calibration, risk reclassification, or treatment-response stratification. Without such evidence, HMB should remain a biological hypothesis rather than progress to complex scoring or interventional testing.

### 6.2 Phase 2: external replication and mechanistic validation

The second phase should test whether HMB phenotypes and interaction patterns are externally reproducible and mechanistically plausible. External validation should be performed in independent cohorts using the same predefined endocrine variables, retinal phenotypes, and conventional DR covariates. The goal is to determine whether axis-specific patterns show consistent direction, acceptable calibration, and transportability across populations, diabetes type, sex, age, ancestry, and disease stage. OCT and OCTA are central in this phase because they can separate edema-dominant, ischemic, vascular, and neurodegenerative phenotypes. Candidate endpoints may include inner retinal thickness, ganglion cell-inner plexiform layer measures, central macular thickness, edema morphology, vessel density, foveal avascular zone size, capillary nonperfusion, and injection burden.Mechanistic studies should then test whether the observed clinical patterns are biologically credible. Human retinal organoids and microphysiologic retinal systems can support this step because retinal organoids are increasingly used for retinal disease modeling and drug-discovery platforms (56), and recent human retinal organoid work has been used to model cellular and molecular features of DR (57). Blood-retinal barrier-on-chip and ocular organ-on-chip systems may be particularly useful for leakage, endothelial-pericyte interaction, and barrier-resistance questions, and broader organ-on-chip work supports their application in experimental ophthalmology (58). These models can expose retinal systems to high glucose together with IGF-1 alteration, cortisol or aldosterone/MR activation, low T3 conditions, or estradiol withdrawal alone and in factorial combinations. They should determine whether combined endocrine-glucose stress produces additive, supra-additive, antagonistic, or temporally persistent effects on oxidative stress, inflammatory cytokines, VEGF signaling, glial activation, apoptosis, barrier resistance, and epigenetic marks.

These experimental systems should be interpreted as mechanistic support rather than substitutes for clinical outcome validation. Likewise, Mendelian randomization, pharmacogenomics, omics, and machine-learning approaches should be used as auxiliary tools for causal triangulation, target prioritization, and model refinement only after the core variables and phenotypes have shown reproducibility. Advancement beyond Phase 2 should require both external clinical consistency and mechanistic evidence supporting a plausible endocrine-retinal interaction pattern.

### 6.3 Phase 3: biomarker-selected interventional validation

Only after Phase 1 and Phase 2 support a specific endocrine-retinal phenotype should HMB move toward biomarker-selected interventional validation. Intervention is therefore the final validation step, not the starting point of clinical translation. Trials should enroll patients whose retinal phenotype and endocrine profile match the axis being tested, and they should include both retinal endpoints and systemic safety endpoints. The trial question should remain narrow: in this selected phenotype, does modification of this endocrine axis improve a predefined retinal endpoint without unacceptable systemic risk? Genetic and pharmacogenomic tools may help prioritize targets before interventional testing, but they should not be treated as substitutes for clinical or mechanistic validation. Mendelian randomization has been used to identify potential DR biomarkers, and pharmacogenomic approaches have also been applied to DR drug-target discovery (59, 60). In the HMB framework, such tools are most useful when they converge with a reproducible endocrine-retinal phenotype and supportive retinal model evidence. A genetic or pharmacogenomic signal should therefore be viewed as target-prioritization evidence rather than as sufficient justification for intervention.For example, only if an IGF-1-related phenotype is reproducible across clinical and mechanistic studies should an IGF-1-selected interventional trial be considered. Mineralocorticoid receptor-related studies should preferably begin in patients already receiving mineralocorticoid receptor antagonists for approved renal or cardiovascular indications, with retinal endpoints added prospectively. CPAP studies should require objective sleep apnea severity and adherence data. Thyroid-related studies should begin with patients who have defined thyroid phenotypes or standard endocrine indications. Sex steroid-related research should begin with registries and careful observational designs before any hormone intervention is considered. Interventional studies should not be judged only by visual acuity; they should include OCT/OCTA progression, edema recurrence, injection burden, ETDRS step change, retinal neurodegeneration, and systemic adverse events. If a biomarker changes without retinal benefit, or if systemic risk is unacceptable, the matched interventional claim should be rejected.

## 7 Discussion

### 7.1 Strengths of the HMB hypothesis

The main strength of the HMB hypothesis is that it addresses a common gap between standard DR risk assessment and real clinical behavior. HbA1c, blood pressure, diabetes duration, kidney disease, lipid status, and baseline retinopathy grade remain essential predictors of DR onset and progression. However, these variables do not fully explain all patterns seen in practice. Some patients develop rapid progression, recurrent edema, early neurodegeneration, or suboptimal response to standard therapy despite apparently reasonable systemic control. Others show retinal acceleration during puberty, pregnancy, menopause, thyroid dysfunction, sleep disruption, chronic stress, or rapid metabolic change. HMB provides a structured way to study these residual-risk phenotypes without weakening the central role of hyperglycemia.

A second strength is biological coherence. The diabetic retina is not only a passive vascular target of glucose toxicity. It is a neural and vascular tissue with high energy demand, strict barrier regulation, active glial support, and local receptor and enzyme systems. GH/IGF-1 signaling, HPA–glucocorticoid signaling and related GR/MR balance, thyroid hormone signaling, and sex steroid signaling may affect retinal neurons, Müller glia, endothelial cells, pericytes, microglia, and RPE through partly distinct but overlapping pathways. These pathways converge on oxidative stress, inflammation, VEGF signaling, blood-retinal barrier dysfunction, neurodegeneration, and metabolic memory. This convergence makes the HMB framework biologically plausible because it links systemic endocrine variation to retinal injury mechanisms that are already central to DR.

A third strength is that HMB is not built around a single hormone or a single disease stage. This is important because DR is heterogeneous. Early neurodegeneration, persistent DME, ischemic progression, and proliferative disease may share diabetic risk factors but may not be driven by the same active biology in every patient. In one patient, GH/IGF-1 signaling may be most relevant to pubertal proliferative activity or refractory edema. In another, MR-related inflammation, sleep apnea, and cortisol rhythm disturbance may be more relevant to recurrent leakage. In another, low-normal FT3 may reflect reduced metabolic reserve and inflammatory vulnerability. HMB therefore encourages mechanism-based phenotyping rather than treating all DR as one uniform process.

A fourth strength is falsifiability. HMB can be tested and can also be disproven. A prospective cohort can test whether a predefined multidimensional endocrine-retinal profile improves risk prediction beyond standard clinical variables. OCT and OCTA can test whether participants assigned *a priori* to a study-specific higher-burden endocrine-retinal research stratum show faster structural progression, vessel density loss, inner retinal thinning, nonperfusion, or recurrent edema than participants in a corresponding lower-burden stratum with similar standard clinical risk profiles. Treatment-response studies can test whether persistent DME is enriched for specific endocrine-retinal patterns. Human retinal organoids and blood-retinal barrier-on-chip models can test whether combined endocrine and glucose stress produces more injury than single-axis exposure. Biomarker-selected trials can test whether correcting a matched endocrine disturbance changes retinal endpoints. These testable predictions make HMB more than a narrative explanation.

Finally, HMB has practical research value because it starts from the retinal phenotype. It does not ask clinicians to order broad hormone panels without a reason. Instead, it asks whether a specific retinal pattern should prompt a specific endocrine-retinal research question. This phenotype-first approach may help reduce noise in translational DR studies. Many interventions fail because they are tested in broad, mixed populations. HMB supports enrichment. The question is not whether every patient with DR has an endocrine driver. The more useful question is whether selected retinal phenotypes show measurable endocrine-retinal signatures that predict progression, treatment response, or treatment resistance.

### 7.2 Limitations and risks of overinterpretation

Several limitations must be emphasized. First, HMB is a hypothesis and has not yet been validated as a clinical tool. Much of the supporting evidence is observational, preclinical, historical, or derived from adjacent endocrine and retinal conditions. Some studies are small, and some findings are inconsistent. Association should not be interpreted as causation. A single abnormal or low-normal hormone value does not prove that the hormone caused DR progression. Repeated measurements, careful phenotyping, and endocrine expertise are needed before any causal interpretation can be made.

Second, endocrine markers are dynamic. IGF-1 varies with age, puberty, nutritional status, liver function, and diabetes type. Cortisol varies by time of day, sleep quality, psychological stress, medication exposure, depression, and acute illness. Thyroid hormone levels may be affected by systemic inflammation, kidney disease, body composition, and non-thyroidal illness. Sex steroid interpretation depends on age, menstrual status, menopause, pregnancy, hormone therapy, PCOS, obesity, and binding proteins. These variables can easily confound endocrine-retinal analyses. Therefore, HMB studies should avoid overreliance on single time-point serum measurements. Longitudinal sampling and context-specific interpretation are essential.

Third, serum values may not accurately reflect retinal tissue exposure. The retina has local hormone-related mechanisms, including thyroid hormone conversion, corticosteroid receptor balance, IGF-1 pathway routing, and local sex steroid-related signaling. Barrier status may also influence retinal exposure to circulating factors. A patient may have a normal systemic hormone panel but altered retinal receptor expression, enzyme activity, or downstream signaling. This is why HMB validation should combine systemic biomarkers with retinal imaging and experimental models. Blood tests alone are unlikely to define the full endocrine state of the diabetic retina.

Fourth, there is a risk of therapeutic overreach. HMB should not be used to justify broad off-label endocrine treatment. Somatostatin analogs, thyroid hormone, MR antagonists, CPAP, corticosteroids, and menopausal hormone therapy are not interchangeable interventions. Each has its own approved indications, contraindications, systemic risks, and monitoring requirements. Treating biochemical hypothyroidism, managing sleep apnea, or using finerenone for approved renal or cardiovascular indications may be appropriate for systemic care, but using these interventions specifically to prevent or treat DR requires retinal endpoint evidence. HMB should guide research questions, not premature treatment recommendations.

Fifth, HMB may not apply equally to all patients with DR. In many patients, retinal disease is sufficiently explained by long-standing hyperglycemia, hypertension, kidney disease, dyslipidemia, delayed screening, or advanced baseline retinopathy. These patients do not need complex endocrine stratification outside standard care. The potential value of HMB is greatest in residual-risk and atypical phenotypes, such as rapid progression despite reasonable systemic control, recurrent or persistent DME, early neurodegeneration, pubertal proliferative disease, postmenopausal acceleration, thyroid-linked disease, and DR with untreated sleep apnea. A useful HMB model must show when endocrine stratification adds value and when it does not.

Finally, HMB must meet a high predictive standard. It is not enough for a hormone marker to be statistically associated with DR. To become clinically or translationally useful, a predefined HMB research profile must improve prediction beyond existing variables such as age, diabetes duration, HbA1c, blood pressure, kidney function, lipid status, and baseline DR severity. It should improve discrimination, calibration, risk reclassification, treatment-response stratification, or mechanistic trial enrichment. If HMB does not add measurable value beyond standard models, it should remain a biological concept rather than a clinical framework.

### 7.3 Implications for future endocrine-retinal research

If validated through the phased roadmap described above, HMB may support closer collaboration between retina specialists and endocrinologists in selected research-defined phenotypes. This collaboration should remain phenotype-first and research-oriented. The retinal phenotype defines the research question, endocrine expertise supports contextual interpretation of axis-specific measurements, and the protocol prospectively follows both retinal and systemic outcomes. The broader implication is that DR research may need to move beyond disease grading alone toward mechanism-based phenotyping. Traditional categories such as no DR, NPDR, PDR, and DME are clinically useful, but they do not fully describe active biology. A patient with recurrent inflammatory edema, a patient with early neurodegeneration, and a patient with rapidly progressive ischemia may require different research questions even if their clinical grade overlaps. This future research should remain conservative in clinical interpretation. Future research should therefore use HMB as an enrichment framework. The phenotype comes first, the endocrine signal is then tested for added value, and the retinal endpoint is chosen to match the mechanism.

## 8 Conclusion

The diabetic retina should be understood as an endocrine-responsive neurovascular tissue rather than only a downstream vascular target of hyperglycemia. In this framework, GH/IGF-1 signaling, HPA–glucocorticoid signaling and related GR/MR balance, thyroid hormone signaling, and sex steroid signaling may modify retinal vulnerability through receptor-mediated signaling, local hormone metabolism, glial and vascular responses, mitochondrial reserve, and blood-retinal barrier regulation. The HMB hypothesis proposes that these axis-specific signals can interact with hyperglycemia, blood pressure, kidney disease, lipid status, and baseline retinal injury to amplify oxidative stress, inflammation, VEGF signaling, blood-retinal barrier dysfunction, neurodegeneration, and metabolic memory. HMB is not proposed as a clinical screening tool or as a basis for broad endocrine treatment in patients with diabetic retinopathy. Its value is as a research-oriented framework for explaining residual retinal risk and treatment heterogeneity in selected phenotypes, including rapid nonproliferative diabetic retinopathy, persistent DME with suboptimal anti-VEGF response, pubertal or young-onset proliferative disease, postmenopausal acceleration, thyroid-linked retinal vulnerability, and diabetic retinopathy with sleep apnea. The hypothesis is useful only if it remains testable. Future studies should determine whether phenotype-matched endocrine-retinal profiling improves prediction beyond standard DR risk factors and whether axis-specific biomarkers are associated with OCT/OCTA progression, recurrent edema, retinal neurodegeneration, or treatment burden. Validation should proceed through a phased pathway: first, prospective repeated-measures endocrine-retinal cohorts should test reproducibility and incremental prediction beyond standard DR variables; second, independent cohorts and retinal model systems should test external validity and mechanistic plausibility; and only then should biomarker-selected interventional studies evaluate matched retinal endpoints and systemic safety. If validated, HMB may provide a structured way to move part of DR research earlier, from treatment of established retinal complications toward identification of endocrine-retinal vulnerability. It may also support closer collaboration between retina specialists and endocrinologists in selected research-defined phenotypes and help build a more complete, mechanism-based view of the diabetic retinal environment.

## Data Availability

The original contributions presented in the study are included in the article/supplementary material. Further inquiries can be directed to the corresponding author.
